# Chaperone Spy Protects Outer Membrane Proteins from Folding Stress via Dynamic Complex Formation

**DOI:** 10.1128/mBio.02130-21

**Published:** 2021-10-05

**Authors:** Wei He, Gangjin Yu, Tianpeng Li, Ling Bai, Yuanyuan Yang, Zixiao Xue, Yonghao Pang, Dana Reichmann, Sebastian Hiller, Lichun He, Maili Liu, Shu Quan

**Affiliations:** a State Key Laboratory of Bioreactor Engineering, East China University of Science and Technology, Shanghai Collaborative Innovation Center for Biomanufacturing (SCICB), Shanghai, China; b State Key Laboratory of Magnetic Resonance and Atomic and Molecular Physics, Wuhan National Laboratory for Optoelectronics, National Center for Magnetic Resonance in Wuhan, Key Laboratory of Magnetic Resonance in Biological Systems, Innovation Academy for Precision Measurement Science and Technology, Chinese Academy of Sciences, Wuhan, China; c Department of Biological Chemistry, The Alexander Silberman Institute of Life Sciences, Safra Campus Givat Ram, The Hebrew University of Jerusalem, Jerusalem, Israel; d Biozentrum, University of Basel, Basel, Switzerland; e University of Chinese Academy of Sciences, Beijing, China; University of Michigan–Ann Arbor

**Keywords:** chaperone, outer membrane protein biogenesis, folding stress, protein-protein interaction, nuclear magnetic resonance spectroscopy

## Abstract

Gram-negative bacteria have a multicomponent and constitutively active periplasmic chaperone system to ensure the quality control of their outer membrane proteins (OMPs). Recently, OMPs have been identified as a new class of vulnerable targets for antibiotic development, and therefore a comprehensive understanding of OMP quality control network components will be critical for discovering antimicrobials. Here, we demonstrate that the periplasmic chaperone Spy protects certain OMPs against protein-unfolding stress and can functionally compensate for other periplasmic chaperones, namely Skp and FkpA, in the Escherichia coli K-12 MG1655 strain. After extensive *in vivo* genetic experiments for functional characterization of Spy, we use nuclear magnetic resonance and circular dichroism spectroscopy to elucidate the mechanism by which Spy binds and folds two different OMPs. Along with holding OMP substrates in a dynamic conformational ensemble, Spy binding enables OmpX to form a partially folded β-strand secondary structure. The bound OMP experiences temperature-dependent conformational exchange within the chaperone, pointing to a multitude of local dynamics. Our findings thus deepen the understanding of functional compensation among periplasmic chaperones during OMP biogenesis and will promote the development of innovative antimicrobials against pathogenic Gram-negative bacteria.

## INTRODUCTION

The outer membrane (OM) of Gram-negative bacteria is the first barrier that controls nutrient uptake and protects cells from diverse environmental stresses. Outer membrane proteins (OMPs) embedded in the OM have a characteristic β-barrel fold and exert various functions, including nutrient transport, cell adhesion, virulence, and multidrug resistance (1, 2). Before adopting their functional states in the OM, OMP precursors must first traverse the hydrophobic inner membrane and then pass through the aqueous periplasmic space before being properly inserted into the OM and folding correctly (3). Understanding the biogenesis of OMPs enables the design of new antibiotics that interfere with these processes (4), thus providing new strategies for combating various pathogenic bacteria, such as Pseudomonas aeruginosa and Salmonella enterica.

**FIG 1:**
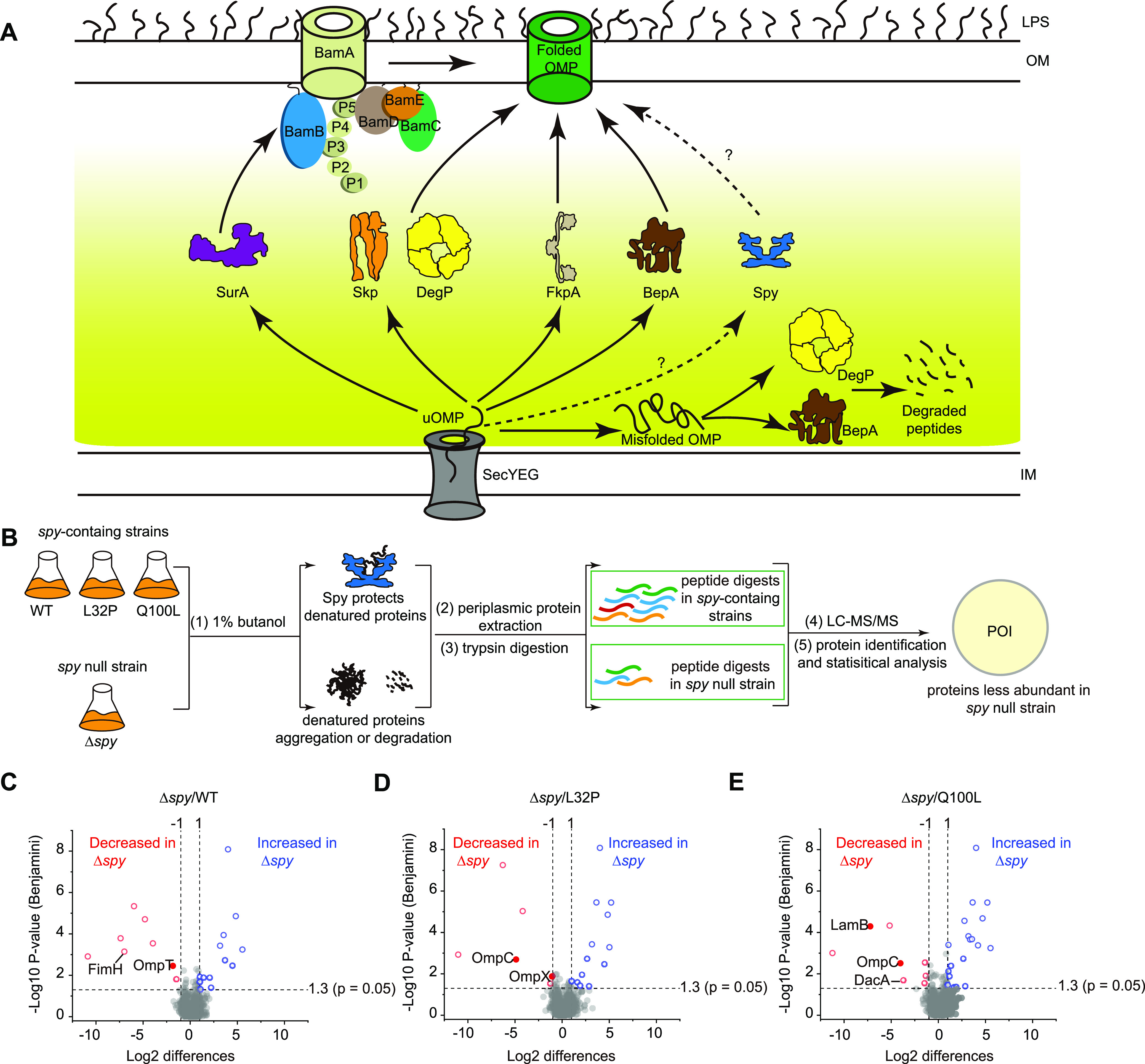
Comparative proteomic analyses of *spy*-containing and *spy*-null strains under butanol stress links Spy to outer membrane protein (OMP) biogenesis. (A) Cartoon representation of the chaperone networks of OMP biogenesis in the periplasm. The OMP chaperones SurA, Skp, DegP, FkpA, and BepA bind unfolded OMPs (uOMPs) translocated from the SecYEG translocase and assist in their correct folding on the OM. SurA was previously shown to deliver uOMPs to the BAM complex for folding and membrane insertion, but whether other molecular chaperones can deliver uOMPs to the BAM complex remains unclear. DegP and BepA can also serve as proteases to degrade misfolded OMPs. (B) Schematic illustration of the comparative proteomics workflow used to identify Spy’s potential client proteins. The wild-type (WT) and Δ*spy* strains, together with two other *spy*-containing strains (L32P and Q100L) were cultivated to the mid-log phase, treated with 1% butanol for 1.5 h, and then osmotically shocked to obtain the cell envelope fractions. Protein abundance was measured following trypsin digestion, liquid chromatography-tandem mass spectrometry (LC-MS/MS), protein identification, and quantitative analysis. Proteins that are less abundant in the *spy*-null strain were our proteins of interest (POI) for further analyses. (C to E) Volcano plots showing pairwise comparisons of differences in protein abundance between WT and Δ*spy* (C), L32P and Δ*spy* (D), and Q100L and Δ*spy* (E) strains. Significantly different proteins are colored in either red (decreased upon the *spy* deletion) or blue (increased upon the *spy* deletion), according to a *P* value of 0.05 and a fold change of >2. The volcano plot is related to Data Set S2 in the supplemental material. Significantly decreased OMPs in the *spy* null strain are highlighted by the filled circles, and all significantly decreased envelope proteins are labeled.

Bacteria employ a network of molecular chaperones and membrane-embedded proteins to facilitate OMP biogenesis (5). OMP precursors are routed into the periplasm by the Sec protein translocation pathway and are then escorted by various periplasmic chaperones to the β-barrel assembly machinery (Bam) for OM insertion (Fig. 1A). Multiple achievements in Bam structural and functional biology have informed several theories about BAM-mediated OMP biogenesis, collectively presenting a fascinating picture about the nature of the insertion and folding processes for OMPs in the OM (6). However, there is little empirical support for many of the mechanistic details about chaperone-mediated periplasmic shuttling and quality control. It is generally agreed that chaperone binding stabilizes the very hydrophobic transmembrane domains of OMPs, keeping them in a soluble, largely unfolded yet folding-competent state (7, 8). The three most thoroughly characterized periplasmic chaperones are survival protein A (SurA), seventeen-kilodalton protein (Skp), and DegP, which also functions as a protease. SurA has been shown to exert major facilitation roles for OMP biogenesis, while Skp and DegP are known to function in parallel as part of a quality control network that rescues OMPs which “fall off” the SurA pathway, an outcome that is especially prevalent when cells are experiencing stress conditions (9). Other factors that have been implicated in the biogenesis of specific OMP targets include the periplasmic chaperone FkpA and the protease BepA (10, 11) (Fig. 1A).

10.1128/mBio.02130-21.9 DATA SET S2 List of proteins with significantly different abundances in the *spy*-containing strains compared to those in the *spy*-null strain. Download Data Set S2, XLSX file, 0.2 MB. Copyright © 2021 He et al. 2021 He et al. https://creativecommons.org/licenses/by/4.0/ This content is distributed under the terms of the Creative Commons Attribution 4.0 International license.

Many periplasmic chaperones are promiscuous in their substrate binding and apparently function in redundant roles, and multiple studies have investigated whether other periplasmic chaperones can also contribute to OMP biogenesis (12–14). The chaperone Spy was originally discovered in a genetic screen to stabilize the bacterial immunity protein 7 (Im7) in the periplasm (15), and several Spy mutants, including the Spy^Q100L^ and Spy^L32P^ variants, were found to have tighter binding to client proteins than that of wild-type Spy (16). Experiments using the client proteins Im7 and Fyn SH3 have advanced the understanding of Spy’s chaperone mechanism *in vitro* (17–20), yet much remains unknown about the physiological function(s) of Spy. It is known that *spy* deletion results in activation of the σ^E^ stress response pathway, which monitors OMP biogenesis and can be activated by accumulation of misfolded OMPs upon cell envelope damage or excessive OMP synthesis, implicating that *spy* deletion may have an influence on OMP homeostasis (21, 22).

Here, by combining comparative proteomics with both phenotypic and genetic assays, we found that Spy has a role in the OMP periplasmic quality control network. Using physiological OMP substrates, we showed that Spy was able to interact with unfolded OMPs and inhibit their aggregation *in vitro*. Furthermore, by employing nuclear magnetic resonance (NMR) spectroscopy, we explored the interactions of Spy with OmpX and tOmpA at the atomic level. Our results suggest that Spy can compensate for Skp by acting via a similar binding mode to maintain OMPs in a largely unfolded state. However, unlike Skp, which is generally thought to act as a holding chaperone, Spy supports the formation of local secondary structures in the OMPs it binds. Overall, our discovery of a new role and mechanism for Spy in assisting OMP biogenesis enables a more comprehensive understanding of the OMP quality control network and will inspire the development of innovative antibiotics targeting OMP biogenesis.

## RESULTS

### Comparative proteomics under butanol stress identified OMPs as potential Spy clients.

Many stress-activated chaperones act as the first line of defense to protect cells against conditions that cause sudden protein unfolding and aggregation (23). Therefore, conducting comparative proteomic profiling of chaperone null strains under protein unfolding-inducing conditions can often provide clues about the physiological function(s) of the particular chaperone proteins (24). Butanol is a protein unfolding agent that also massively induces Spy production (16, 25). We thus performed periplasmic proteomic profiling of *spy*-containing and *spy*-null strains under 1% butanol stress on the basis of the Escherichia coli K-12 MG1655 strain. We reasoned that proteins which are strictly dependent on Spy for their proper folding would be incorrectly folded in the absence of the chaperone under butanol stress and would therefore be more prone to proteolysis or aggregation, which would result in decreased accumulation levels in *spy*-null cells.

We extracted the cell envelope fractions of mid-log-phase cells from WT and Δ*spy* cells, as well as from two additional *spy*-containing strains harboring a single-copy gene of the L32P or Q100L activity-enhancing Spy variant on the chromosome (here referred to as L32P and Q100L; see detailed strain information in Table 1), under the native *spy* promoter. Proteins from three biological replicates of each sample group were extracted, digested by trypsin, alkylated, and analyzed by liquid chromatography-tandem mass spectrometry (LC-MS/MS) (see detailed methods in Text S1 in the supplemental material) (Fig. 1B). To identify and quantify changes in protein abundance between the four strains, we applied label-free quantification (LFQ) using MaxQuant software (26) (see detailed methods in Text S1). We identified 1,023 proteins in total that appeared at least in one strain in all three replicates (Data Set S1). To identify proteins affected by the *spy* depletion, we applied a stringent two-tailed *t* test analysis (*P* < 0.05) to identify significantly decreased and increased proteins in WT, L32P, and Q100L relative to those to the Δ*spy* strain (Fig. 1C to E and Table 2, and Table S1). Among all of the identified proteins, 25 and 17 are significantly increased or decreased, respectively, in the Δ*spy* strain. Proteins with higher levels in the Δ*spy* strain are localized in the cytoplasm, peripheral inner membrane, or nucleoid, and are involved in metabolic processes, indicating that the absence of Spy affects basal metabolism (Fig. 1C to E and Table 2, and Table S1).

**TABLE 1:** Strains and plasmids used in this work

Strain or plasmid	Genotype and/or relevant description^*a*^	Source or reference no.
E. coli strains		
WT (MG1655, Δ*hsdR*)	F^−^ λ^−^ *ilvG*^−^ *rfb-50* *rph-1* Δ*hsdR*	Lab stock
Δ*spy*	F^−^ λ^−^ *ilvG*^–^ *rfb-50* *rph-1* Δ*hsdR* Δ*spy*	Lab stock
Q100L	F^−^ λ^−^ *ilvG*^−^ *rfb-50* *rph-1* Δ*hsdR spy-Q100L rpsL150*	This study
L32P	F^−^ λ^−^ *ilvG*^−^ *rfb-50* *rph-1* Δ*hsdR spy-L32P rpsL150*	This study
Δ*skp*	F^−^ λ^−^ *ilvG*^−^ *rfb-50* *rph-1* Δ*hsdR* Δ*skp*	This study
Δ*fkpA*	F^−^ λ^−^ *ilvG*^−^ *rfb-50* *rph-1* Δ*hsdR* Δ*fkpA*	This study
Δ*skp* Δ*fkpA*	F^−^ λ^−^ *ilvG*^−^ *rfb-50* *rph-1* Δ*hsdR* Δ*skp* Δ*fkpA*	This study
Δ*skp* Δ*fkpA* Δ*spy*	F^−^ λ^−^ *ilvG*^−^ *rfb-50* *rph-1* Δ*hsdR* Δ*skp* Δ*fkpA* Δ*spy*	This study
Δ*skp* Δ*fkpA baeS-E264K*	F^−^ λ^−^ *ilvG*^−^ *rfb-50* *rph-1* Δ*hsdR* Δ*skp* Δ*fkpA baeS-E264K*	This study
Δ*skp* Δ*fkpA* Δ*spy baeS-E264K*	F^−^ λ^−^ *ilvG*^−^ *rfb-50* *rph-1* Δ*hsdR* Δ*skp* Δ*fkpA* Δ*spy baeS-E264K*	This study
BL21(DE3)	F^−^ *ompT* *gal* *dcm* *lon* *hsdS_B_*(*r_B_*^−^*m_B_*^−^) λ(DE3 [*lacI* *lacUV5*-*T7p07* *ind1* *sam7* *nin5*]) [*malB*^+^]_K-12_(λ^S^)	Lab stock
Plasmids		
pET28b(+)	Kan^r^; for inducible production of recombinant protein in E. coli	Lab stock
pET28b(+)-HisSUMO-*spy*	Kan^r^; for purification of Spy from E. coli	16
pET28b(+)-HisSUMO-*spy-Q100L*	Kan^r^; for purification of Spy^Q100L^ from E. coli	16
pET28b(+)-HisSUMO-*skp*	Kan^r^; for purification of Skp from E. coli	This study
pET28b(+)-His-*ompX*	Kan^r^; for purification of OmpX from E. coli; used in the intrinsic tryptophan fluorescence assay and aggregation assay	This study
pET28b(+)-His-*ompC*	Kan^r^; for purification of OmpC from E. coli; used in the intrinsic tryptophan fluorescence assay and aggregation assay	This study
pET28b(+)-His-*ompT*	Kan^r^; for purification of OmpT from E. coli; used in the intrinsic tryptophan fluorescence assay and aggregation assay	This study
pET28b(+)-His-*ompA*	Kan^r^; for purification of OmpA from E. coli; used in the intrinsic tryptophan fluorescence assay and aggregation assay	This study
pET28b(+)-His-*lamB*	Kan^r^; for purification of LamB from E. coli; used in the intrinsic tryptophan fluorescence assay and aggregation assay	This study
pET28b(+)-*tompA*	Kan^r^; for purification of the transmembrane domain of OmpA from E. coli; used in NMR experiments	This study
pET28b(+)-*ompX*	Kan^r^; for purification of untagged OmpX from E. coli; used in NMR experiments	This study
pCDFTrc	Kan^r^; vector-only control; used in spot titer experiments and Western blot experiments	This study
pCDFTrc-*Spy*	Kan^r^; for expression of Spy; used in spot titer experiments and Western blot experiments	This study
pCDFTrc-*Spy-Q100L*	Kan^r^; for expression of Spy^Q100L^; used in spot titer experiments and Western blot experiments	This study
pCDFTrc-*Spy-L32P*	Kan^r^; for expression of Spy^L32P^; used in spot titer experiments and Western blot experiments	This study

a NMR, nuclear magnetic resonance.

**TABLE 2:** Summary of proteins with a decreased abundance in the *spy* null strain

Protein	Fold change (×)	*P*	Identified comparison group	Topology class^*a*^	Description
OmpC	29.6	0.002	L32P/*Δspy*	Outer membrane β-barrel protein	Outer membrane pores allowing for small molecular diffusion
	16.3	0.003	Q100L/*Δspy*	Outer membrane β-barrel protein	Outer membrane pores allowing for small molecular diffusion
OmpX	2.1	0.013	L32P/*Δspy*	Outer membrane β-barrel protein	Related to type 1 fimbriae production; biofilm formation and invasion of pathogenic E. coli
OmpT	3.6	0.004	WT/*Δspy*	Outer membrane β-barrel protein	Protease
DacA	12.9	0.021	Q100L/*Δspy*	Periplasmic protein	Carboxypeptidase involved in peptidoglycan biosynthesis
YaeQ	2.7	0.028	Q100L/*Δspy*	Cytoplasmic protein	Uncharacterized
YeeN	2.7	0.003	Q100L/*Δspy*	Cytoplasmic protein	Regulation of transcription
CopA	2.5	0.013	Q100L/*Δspy*	Integral inner membrane protein	Copper-exporting P-type ATPase
PpiB	1.9	0.016	Q100L/*Δspy*	Peripheral inner membrane protein facing the cytoplasm	Peptidyl-prolyl *cis*-*trans* isomerase
MsrB	2.4	0.030	L32P/*Δspy*	Cytoplasmic protein	Peptide methionine sulfoxide reductase
YkgE	2.8	0.016	WT/*Δspy*	Cytoplasmic protein	Uncharacterized
LamB^*c*^	149.6^*b*^	5.01E-05	Q100L/*Δspy*	Outer membrane β-barrel protein	Maltose and maltodextrins transportation
FimH^*c*^	127.0^*b*^	0.001	WT/*Δspy*	Fimbrium	Regulation of length and mediation of adhesion of type 1 fimbriae
SufA^*c*^	78^*b*^	5.54E-08	L32P/*Δspy*	Peripheral inner membrane protein facing the cytoplasm	Iron-sulfur cluster assembly
PmrD^*c*^	167.8^*b*^	1.63E-04	WT/*Δspy*	Cytoplasmic protein	Mediate transcriptional induction of BasR-activated genes to induce polymyxin resistance
YjjX^*c*^	62.4^*b*^	4.51E-06	WT/*Δspy*	Cytoplasmic protein	Inosine/xanthosine triphosphatase
YggU^*c*^	15.6^*b*^	2.85E-04	WT/*Δspy*	Cytoplasmic protein	Uncharacterized
SufE^*c*^	27.9^*b*^	1.96E-05	WT/*Δspy*	Peripheral inner membrane protein facing the cytoplasm	Cysteine desulfuration
	18.2^*b*^	9.31E-06	L32P/*Δspy*		
	35.9^*b*^	4.64E-05	Q100L/*Δspy*		

a The topology class of each protein was noted according to subcellular topology and localization of the Escherichia coli polypeptides (STEPdb).

b The missing label-free quantification (LFQ) values were imputed using deterministic minimal imputation (58), as described in the supplemental materials and methods in Text S1.

c Fold change absent in *Δspy*.

10.1128/mBio.02130-21.5 TABLE S1 Summary of proteins with decreased abundances in the *spy*-containing strains. Download Table S1, DOCX file, 0.02 MB. Copyright © 2021 He et al. 2021 He et al. https://creativecommons.org/licenses/by/4.0/ This content is distributed under the terms of the Creative Commons Attribution 4.0 International license.

10.1128/mBio.02130-21.7 TEXT S1 Supplemental materials and methods for mass spectrometry. Download Text S1, DOCX file, 0.02 MB. Copyright © 2021 He et al. 2021 He et al. https://creativecommons.org/licenses/by/4.0/ This content is distributed under the terms of the Creative Commons Attribution 4.0 International license.

10.1128/mBio.02130-21.8 DATA SET S1 List of all proteins identified in the *spy*-null and *spy*-containing strains. Download Data Set S1, XLSX file, 0.2 MB. Copyright © 2021 He et al. 2021 He et al. https://creativecommons.org/licenses/by/4.0/ This content is distributed under the terms of the Creative Commons Attribution 4.0 International license.

Since Spy functions in the periplasm, we mainly focused on the envelope proteins that were less abundant in the Δ*spy* strain. Notably, we found that *spy* deletion resulted in reduced abundance (or absence) of six envelope proteins, four of which are outer membrane proteins, namely, OmpC, OmpX, OmpT, and LamB (Fig. 1C to E and Table 2). By comparing protein levels between Spy variant-expressing strains and the wild-type strain, we also noted that two envelope proteins, OmpC and DacA (a periplasmic protein involved in peptidoglycan biosynthesis), were more abundant in Spy variant-expressing strains (Table S2). We then focused on the four OMPs and ruled out the possibility that the differential abundance of these OMPs were due to transcriptional variance by comparing their mRNA levels in WT, Δ*spy*, L32P, and Q100L strains (Fig. S1). Together, these results provide a clue that Spy is involved in protecting certain OMPs from denaturation and degradation.

10.1128/mBio.02130-21.1 FIG S1 Quantitative reverse transcription-PCR (RT-PCR) analyses of OmpC, OmpX, OmpT, and LamB mRNA levels in *spy*-containing strains relative to those in the Δ*spy* strain. The mRNA samples were extracted from mid-log-phase cells treated with 1% (vol/vol) butanol for 1.5 h. The maximal values for the mRNA levels of the 4 outer membrane proteins (OMPs) in *spy*-containing strains (wild type [WT], L32P, and Q100L) are less than 1.5-fold the mRNA levels in the Δ*spy* strain, which is insufficient to entirely explain the increase of OMP protein levels in *spy*-containing strains (>2.1-fold). All results are expressed as the mean ± standard deviation (SD); individual data points are derived from 3 biological samples, each with 3 technical repeats. Download FIG S1, PDF file, 0.4 MB. Copyright © 2021 He et al. 2021 He et al. https://creativecommons.org/licenses/by/4.0/ This content is distributed under the terms of the Creative Commons Attribution 4.0 International license.

10.1128/mBio.02130-21.6 TABLE S2 Summary of proteins with significantly different abundances in the L32P and Q100L strains compared to those in the wild-type (WT) strain. Download Table S2, DOCX file, 0.02 MB. Copyright © 2021 He et al. 2021 He et al. https://creativecommons.org/licenses/by/4.0/ This content is distributed under the terms of the Creative Commons Attribution 4.0 International license.

### Deletion of *spy* results in decreased OM integrity.

**FIG 2:**
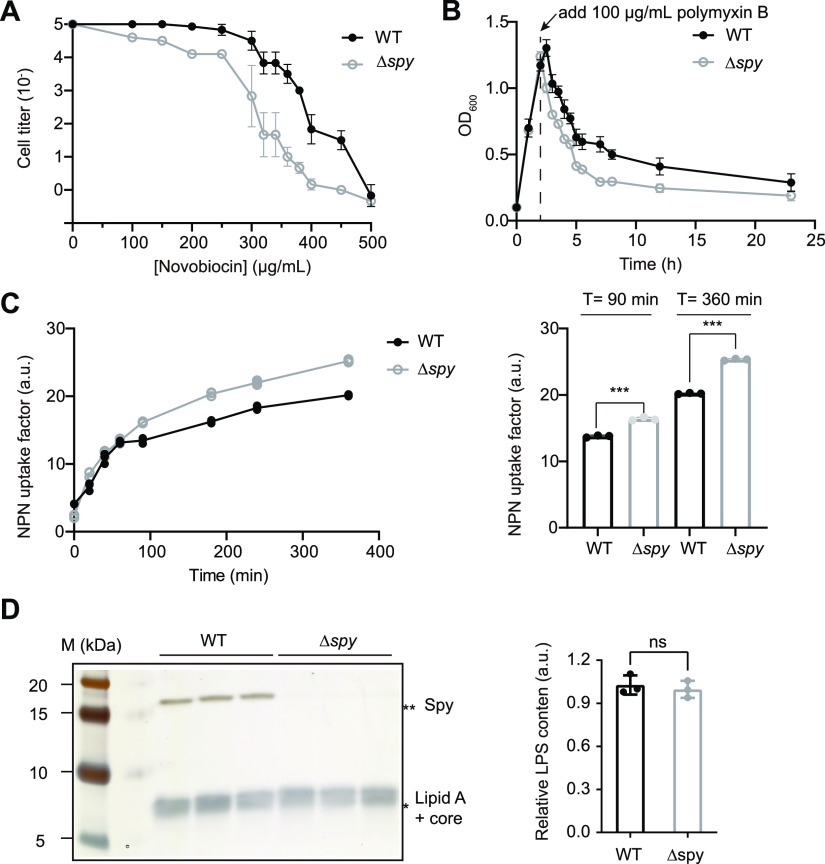
Deletion of *spy* causes OM defects. (A) Susceptibility test of the WT or Δ*spy* strains for cellular uptake of the antibiotic novobiocin. Mid-log-phase cells were serially diluted onto LB plates containing various concentrations of novobiocin. After overnight incubation, cell titers (the maximal cell dilutions that allow growth) were plotted against various concentrations of novobiocin. Mean ± standard deviation (SD) of 3 biological replicates. (B) Polymyxin B susceptibility test of the WT or Δ*spy* strains. Polymyxin B (100 μg/ml) was added to late-log-phase cultures of the WT or Δ*spy* strains, and the optical densities were monitored over time. Mean ± SD of 3 biological replicates. (C) 1-*N*-phenylnaphthylamine (NPN) uptake assay to detect membrane leakage of the WT and Δ*spy* strains under butanol stress. WT or Δ*spy* mid-log-phase cells were treated with 1% butanol. At each of the indicated sampling time points, 0.5 optical density (OD) cells were collected, mixed with 10 μM NPN, and assayed for NPN fluorescence (excitation [*E_x_*] = 350 nm; emission [*E_m_*] = 407 nm). NPN uptake factors were calculated as described in Materials and Methods, and are here plotted against time in the left panel. Individual data points of 3 biological replicates are shown. (Right) NPN uptake factors of the WT and Δ*spy* strains upon 1% butanol stress for 90 min and 360 min were compared. The higher levels of NPN uptake factors in the Δ*spy* strain (mean ± SD, *n* = 3, individual data points are shown; unpaired two-tailed Student’s *t* test; ***, *P* < 0.001) suggest greater membrane leakage in the Δ*spy* strain. (D) Comparison of the LPS content of the WT and *Δspy* strains upon 1% butanol stress. In the left panel, WT and *Δspy* mid-log phase cells were exposed to 1% butanol for 1.5 h, followed by extraction of lipopolysaccharide (LPS) from the two samples, SDS-PAGE, and visualization by silver staining. (Right) The LPS contents of the WT and Δ*spy* strains were compared after quantification by ImageJ. The mean of three WT samples was set to 1.0, and all of the samples were referenced to that value. Deletion of *spy* caused no obvious difference in the extent of LPS biogenesis (mean ± SD, *n* = 3, individual data points are shown; unpaired two-tailed Student’s *t* test; ns, not significant).

Deficiency in OMP biogenesis caused by deletion of known OMP chaperones like SurA and Skp is usually accompanied by increased permeability of the OM (27, 28). Spy’s role in maintaining the level of four OMPs under butanol stress made us wonder whether *spy* deletion can also result in reduced OM integrity. Pursuing this, we examined the resistance of the Δ*spy* and WT strains toward two antibiotics, novobiocin and polymyxin B. Novobiocin is a bulky, DNA gyrase-binding molecule that can hardly cross the cell membrane of Gram-negative bacteria unless the structure of the OM is damaged (29). Polymyxin B is a cationic cyclic peptide that can bind lipopolysaccharide (LPS) and break the OM (30). We found that the Δ*spy* strain showed increased sensitivity toward both novobiocin and polymyxin B, implying more severe OM leakage of the Δ*spy* strain than of the WT strain (Fig. 2A and B).

To further characterize OM permeability changes, we monitored the uptake of a hydrophobic fluorescent probe, 1-*N*-phenylnaphthylamine (NPN) by both Δ*spy* and wild-type strains under butanol stress. NPN only fluoresces weakly in the aqueous environment but fluoresces strongly in phospholipid environment (31). We found that the Δ*spy* strain showed stronger NPN uptake compared with that of the WT, indicating an exacerbated damage in the OM of the Δ*spy* strain (Fig. 2C). The observed OM deficiency phenotypes of the Δ*spy* strain could also result from damage of the LPS rather than OMPs. To examine whether *spy* deletion results in decreased LPS steady-state levels, we extracted LPS components of the wild-type strain and the *Δspy* strain but found no significant difference in their LPS contents under butanol stress (Fig. 2D).

### Spy aids in the biogenesis of certain OMPs when the chaperones Skp and FkpA are deficient.

The observed reduction of several OMPs and decreased OM integrity in the Δ*spy* strain drove us to investigate whether Spy was actively involved in OMP biogenesis. In the past decade, significant progress has been made toward unraveling the pathways and mechanisms of this important event, and many lines of evidence were obtained through multiple-gene knockout and complementation studies (9, 32–34). It was previously shown that Spy overexpression could compensate for the decreased level of an outer membrane protein LptD in the E. coli MC4100 Δ*skp* Δ*fkpA* strain, suggesting that Spy may be part of the LptD biogenesis network (35).

**FIG 3:**
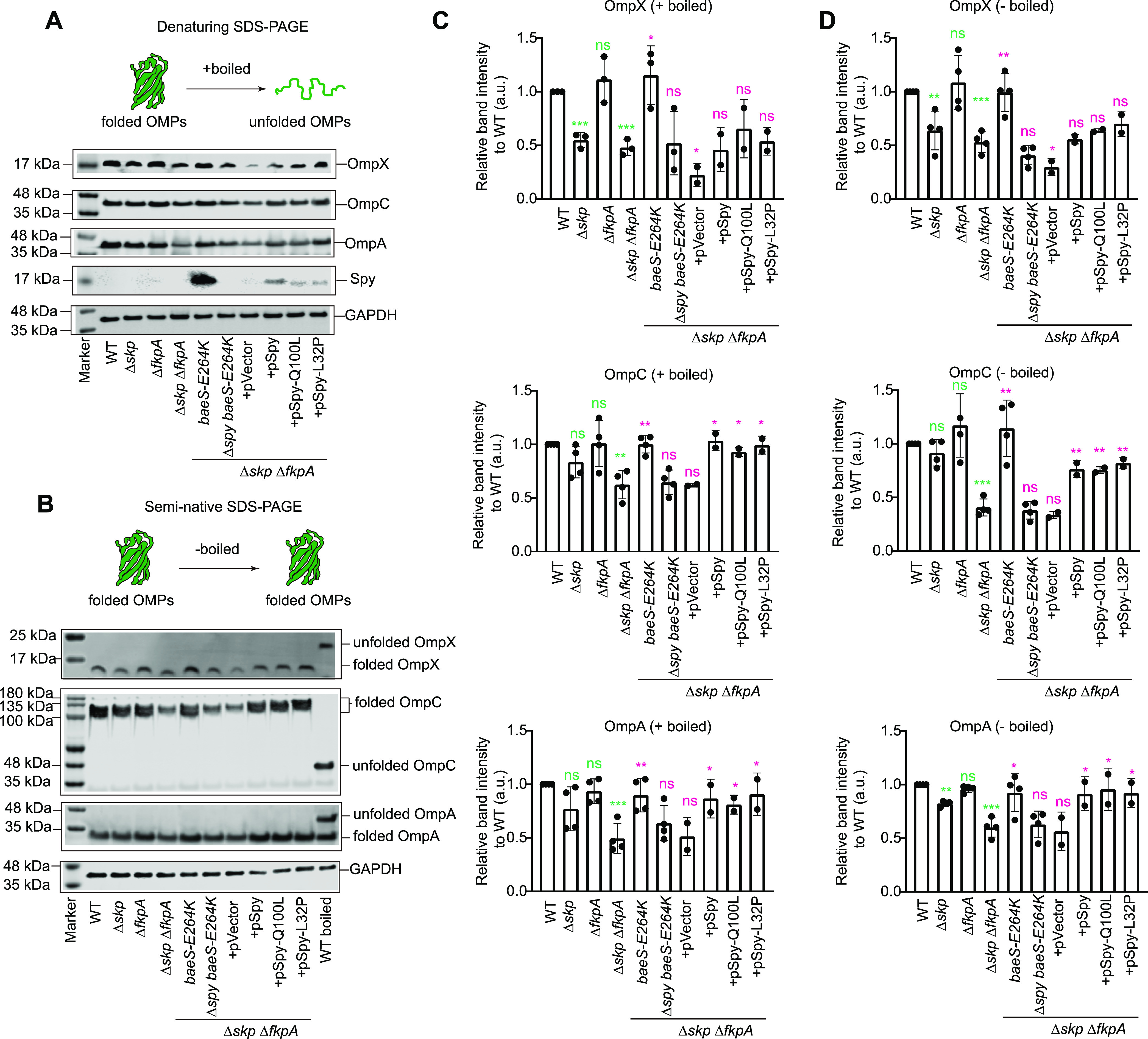
Overexpression of Spy compensates for the reduced OMP levels of the *Δskp ΔfkpA* strain. (A) Immunoblotting of OMPs in the indicated strains using antibodies against OmpC, OmpX, and OmpA. Samples were boiled to denature all proteins before SDS-PAGE. GAPDH was used as a loading control; the blotting against Spy was included to confirm its successful overexpression either from an isopropyl-β-d-thiogalactopyranoside (IPTG)-induced multi-copy plasmid or the constitutively active *baeS-E264K* point mutation on the chromosome of the Δ*skp* Δ*fkpA* strain. (B) Immunoblotting of the folded OMPs in different strains after seminative SDS-PAGE separation. Samples were not boiled, so OMPs were retained in their folded states. The rightmost lane presents unfolded OMPs to support comparisons. GAPDH was used as a loading control. (C, D) Quantifications and comparisons of the OMP steady-state levels in the indicated strains according to the band intensity of Western blots in shown in panels A (C) and B (D). Mean ± SD, *n* = 2 to 4, individual data points are shown. Unpaired two-tailed Student’s *t* test, labels in green and pink represent statistical significances of the OMP steady-state levels of indicated strains compared with the wild-type strain and the *Δskp ΔfkpA* strain, respectively. ns, not significant; *, *P* < 0.05; **, *P* < 0.01; ***, *P* < 0.001.

By SDS-PAGE analyses of whole-cell lysates from multiple chaperone-deleted strains based on E. coli MG1655 (see Table 1 for strain information), we found that deletion of *skp* or *fkpA* alone had little effect on total protein levels, whereas Δ*skp* Δ*fkpA* double knockout resulted in decreased protein intensity in the molecular weight range of OmpC, OmpF, and OmpA (35 to 40 kDa) (Fig. S2A). Overexpression of Spy by introducing a constitutively active *baeS-E264K* point mutation on the chromosome (15) restored the decreased protein level in the 35- to 40-kDa region (Fig. S2A). Western blot analysis results confirmed that *skp fkpA* double deletion led to decreased steady-state levels of OmpX, OmpC, and OmpA, and that Spy overexpression could complement the decreased protein levels of these OMPs in the Δ*skp* Δ*fkpA* strain (Fig. 3A and C). This enhancement was due to Spy’s activity, as deletion of *spy* from the *baeS-E264K* Δ*skp* Δ*fkpA* strain eliminated this effect (Fig. 3A and C). Additionally, in agreement with previously published results (35), we found that Spy was able to maintain the steady-state level of LptD (Fig. S2B).

10.1128/mBio.02130-21.2 FIG S2 Overexpression of Spy partially compensates for the reduced levels of certain OMPs in the *Δskp ΔfkpA* strain. (A) SDS-PAGE analysis of the whole-cell protein components of the wild-type and chaperone deletion strains. Proteins that were deficient in the *Δskp ΔfkpA* strain and were compensated by Spy overexpression were marked with a dashed box. (B, C) Immunoblotting of LptD in various strains. Before immunoblotting with an antibody against LptD, samples boiled with (B) or without (C) β-mercaptoethanol (β-ME) were separated by reducing or nonreducing SDS-PAGE, respectively. Correctly assembled LptD contains two nonconsecutive disulfide bonds and runs more slowly than totally reduced LptD in SDS-PAGE analysis; therefore, the nonreducing SDS-PAGE enables us to detect the levels of correctly assembled LptD in various strains. (D, E) Immunoblotting of OmpT (D) and LamB (E) in the indicated strains. Samples were boiled to denature all proteins before SDS-PAGE. For panels B to E, quantifications and comparisons of OMP steady-state levels in various strains are displayed above the corresponding western blots. Mean ± SD; *n* = 2 to 4; individual data points are shown. Unpaired two-tailed student *t* test; labels in green and pink represent statistical significances of the OMP steady-state levels of the indicated strains compared with the wild-type strain and the *Δskp ΔfkpA* strain, respectively; ns, not significant; *, *P* < 0.05; **, *P* < 0.01; ***, *P* < 0.001. Download FIG S2, PDF file, 2.5 MB. Copyright © 2021 He et al. 2021 He et al. https://creativecommons.org/licenses/by/4.0/ This content is distributed under the terms of the Creative Commons Attribution 4.0 International license.

Furthermore, plasmid overexpression of Spy, Spy^Q100L^, and Spy^L32P^ in the Δ*skp* Δ*fkpA* strain also compensated for the decreased steady-state levels of OmpC and OmpA (Fig. 3A and C). For OmpX and LptD, incorporating the empty vector unexpectedly decreased their steady-state protein levels in the Δ*skp* Δ*fkpA* strain, whereas overexpression of Spy and Spy variants diminished the negative effect of the vector on the levels of these two OMPs (Fig. 3A and C and Fig. S2B and C). Moreover, the folded protein species of these OMPs resolved on seminative SDS-PAGE also revealed Spy-dependent compensation in the Δ*skp* Δ*fkpA* strain (Fig. 3B and D and Fig. S2C), indicating that Spy overexpression not only protects these OMPs from aggregation or degradation but also contributes to the correct folding of these OMPs into native states. We also note results for OmpT and LamB. Although their levels were significantly decreased in Δ*spy* cells treated with butanol (Fig. 1C and E), their levels were not noticeably improved by Spy overexpression in the Δ*skp* Δ*fkpA* strain (Fig. S2D and E).

**FIG 4:**
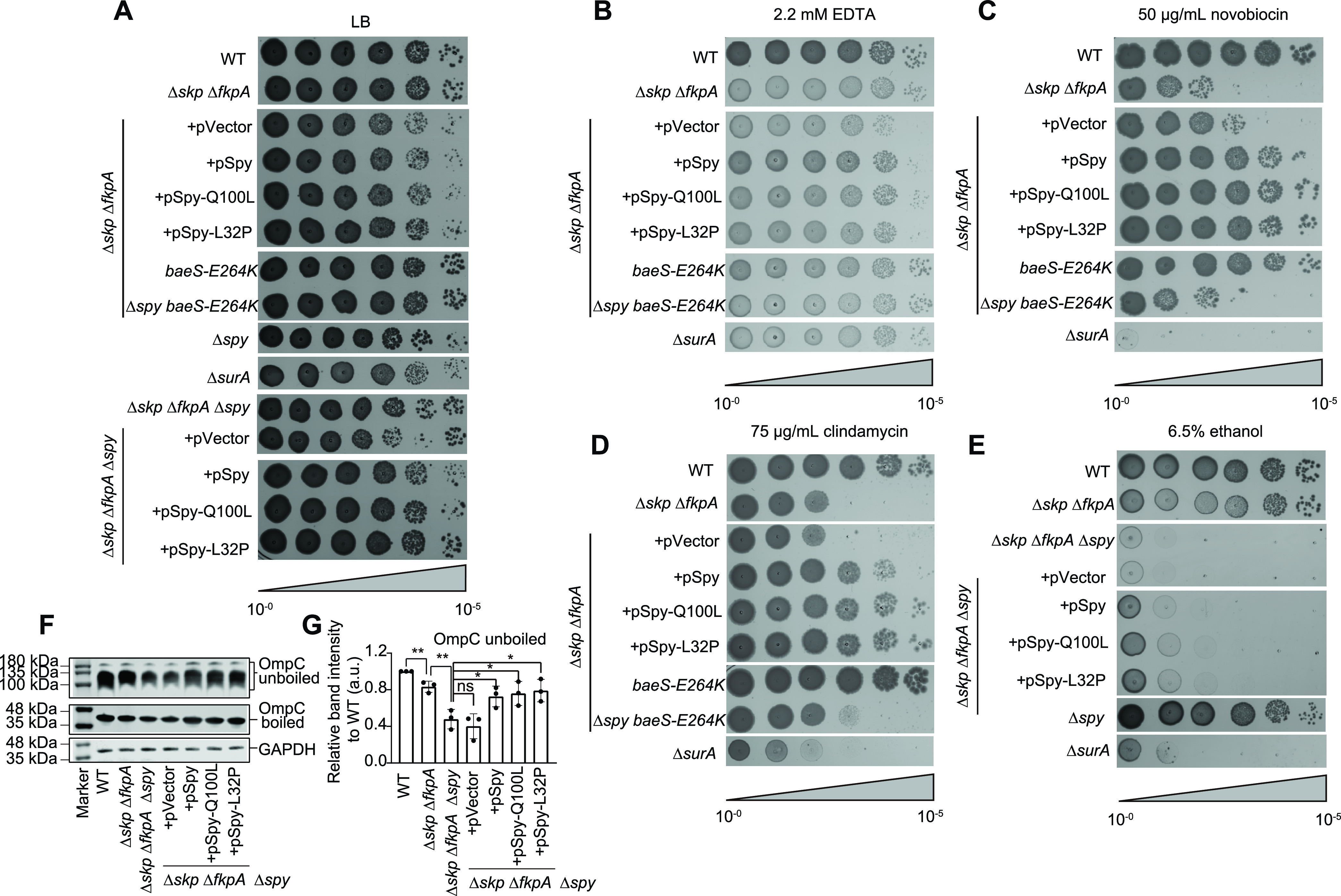
Spy maintains the OM integrity of the Δ*skp* Δ*fkpA* strain and decreases its sensitivities toward specific antibiotics and stress conditions. (A to E) Spot titers of indicated strains on an LB plate (A) or on LB plates supplied with 2.2 mM EDTA (B), 50 μg/ml novobiocin (C), 75 μg/ml clindamycin (D), or 6.5% (vol/vol) ethanol (E), as indicated. Representative pictures of 3 biological replicates are shown. (F and G) Immunoblotting of boiled and unboiled OmpC in the indicated strains using antibodies against OmpC (F). GAPDH was used as a loading control. Cells were grown in LB medium supplied with 2% (vol/vol) ethanol. The steady-state levels of unboiled OmpC in the indicated strains were quantified and compared (G). Mean ± SD, *n* = 3, individual data points are shown. Unpaired two-tailed Student’s *t* test. ns, not significant; *, *P* < 0.05; **, *P* < 0.01; ***, *P* < 0.001.

We next investigated whether overexpression of Spy could compensate for the phenotypes associated with OMP deficiency in the Δ*skp* Δ*fkpA* strain, namely, EDTA and novobiocin sensitivity. Overexpression of Spy and the Spy variants indeed reduced the sensitivity of the Δ*skp* Δ*fkpA* strain toward both EDTA and novobiocin (Fig. 4A to C). To explore the physiological consequences of restoring levels of certain OMPs in the Δ*skp* Δ*fkpA* strain, we then focused on OmpA-and OmpC-specific phenotypes. Deficiency of OmpA but not of other OMPs leads to a 10-fold increase in E. coli susceptibility to clindamycin (36). We found that the Δ*skp* Δ*fkpA* strain with a decreased level of OmpA exhibited sensitivity toward 75 μg/ml clindamycin. Overexpression of Spy and its two variants restored the growth of the Δ*skp* Δ*fkpA* strain almost to the wild-type level (Fig. 4A and D).

Loss of OmpC increases bacterial susceptibility to ethanol stress (36). To our surprise, we only observed a slight increase in ethanol sensitivity of the Δ*skp* Δ*fkpA* strain compared with that of the wild-type strain. Because Spy is massively upregulated under ethanol stress (15) and therefore may protect OmpC in the Δ*skp* Δ*fkpA* strain, we deleted *spy* in the Δ*skp* Δ*fkpA* strain and found a substantial increase in ethanol sensitivity of the triple-knockout strain (Fig. 4A and E). As an important control, Δ*spy* alone had no effect on ethanol sensitivity (Fig. 4E). The increased ethanol sensitivity of the triple-knockout strain could be partially suppressed by overexpression of Spy or its variants (Fig. 4A and E). We also compared the steady-state levels of OmpC in different strains by Western blot analysis (Fig. 4F and G). Quantification of folded OmpC showed a 20% reduction in the Δ*skp* Δ*fkpA* strain and a more than 50% reduction in the triple-knockout strain. Overexpression of Spy and its variants in the triple-knockout strain restored the level of folded OmpC to 70% to 80% of the wild-type level. Taken together, these results suggest that Spy induction by folding stresses such as ethanol may help maintain the protein steady-state levels of certain OMPs that are critical for OM integrity.

### Spy interacts with certain OMPs and inhibits their aggregation.

**FIG 5:**
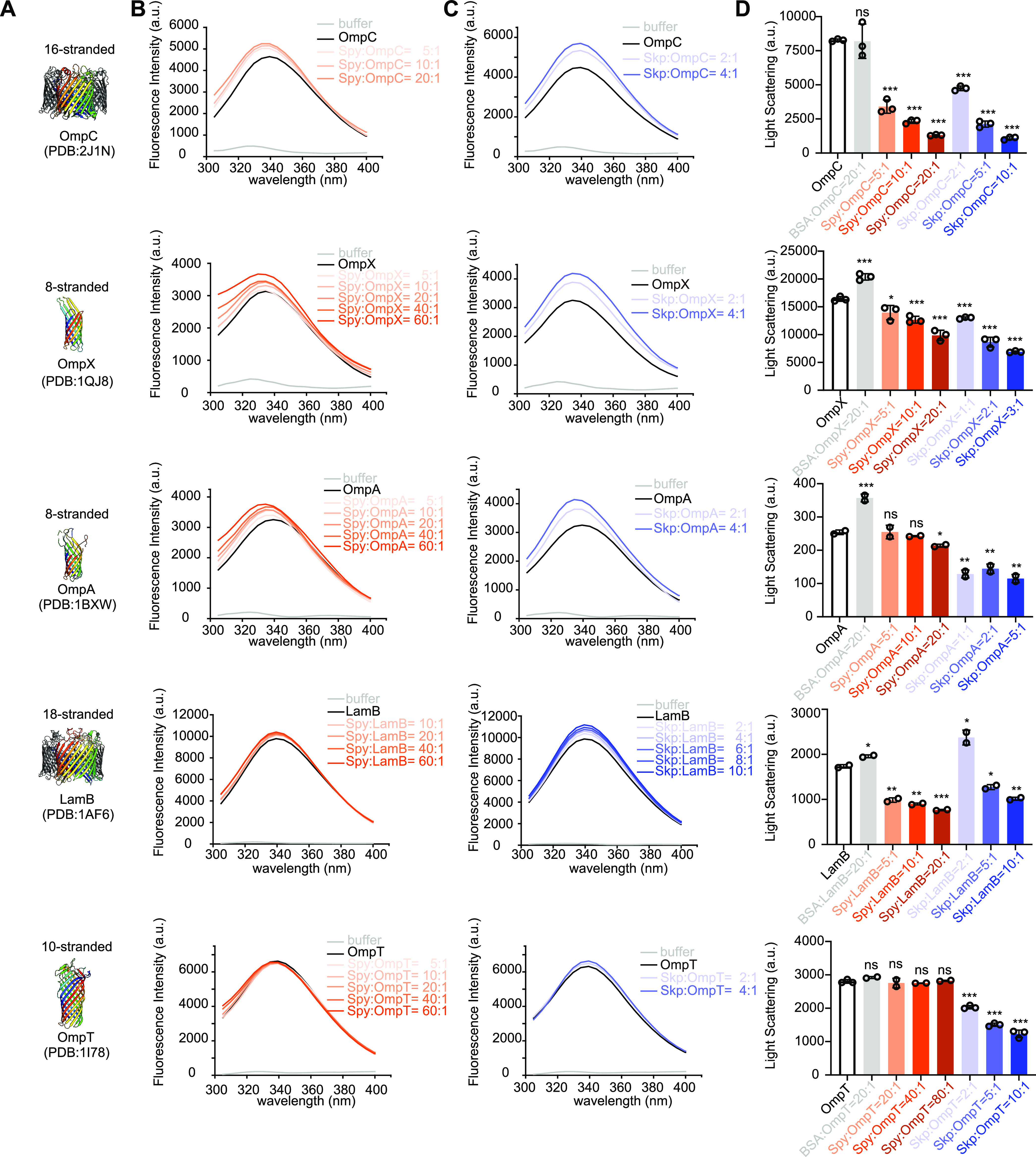
Spy interacts with OMPs and suppresses their aggregation *in vitro*. (A) Cartoon representations of the previously characterized structures of various OMPs used in the tryptophan fluorescence and aggregation assays. (B and C) The indicated OMPs (0.4 μM in 40 mM HEPES, 150 mM NaCl, and 0.24 M urea [pH 7.5]) were titrated with increasing concentrations of Spy (B) and Skp (C). The tryptophan fluorescence emission spectra of OMPs upon Spy addition (B, orange curves) or Skp addition (C, blue curves) were recorded. Titration of Spy into solutions containing OmpX, OmpC, OmpA, or LamB increases the maximal fluorescence intensity and results in a blue shift in the maximal fluorescence wavelength, supporting physical interactions between Spy and these OMPs (B). As a positive control, Skp does undergo interactions with all the tested OMPs (C). Representative curves of 3 independent measurements are displayed. (D) Various OMPs (150 μM in 8 M urea) were diluted 100-fold into 40 mM HEPES-150 mM NaCl (pH 7.5) buffer in the presence or absence of various concentrations of Spy (orange) or Skp (blue). The light scattering signals at 380 nm were recorded. Spy inhibits the aggregation of all the OMPs except OmpT, whereas Skp inhibits aggregation of all OMPs. Mean ± SD, *n* = 2 or 3, individual data points are shown. Unpaired two-tailed Student’s *t* test; labels represent statistical significances of light scattering signals of indicated samples compared with the signal of OMP aggregation alone. ns, not significant; *, *P* < 0.05; **, *P* < 0.01; ***, *P* < 0.001.

Given the multiple lines of *in vivo* evidence suggesting a role for Spy in OMP biogenesis, we next investigated whether Spy can interact physically with these OMPs. With purified OMPs, we measured the changes in their intrinsic tryptophan fluorescence in the presence and absence of Spy to determine whether Spy could directly bind to the unfolded species of these OMPs. Indeed, Spy addition increased the fluorescence intensities, in a dose-dependent manner, for all of the OMPs we tested except OmpT, and also induced blue shifts in the maximal emission wavelengths of OMP fluorescence (Fig. 5A and B).

For comparison, we also measured Skp’s interactions with these OMPs (Fig. 5C) and found similar interacting patterns (i.e., Skp showed strong interactions with OmpC, LamB, OmpA, and OmpX but with not OmpT). However, compared to Spy, noticeably reduced concentrations of Skp were required to shift the fluorescence signals of the same OMPs to the same extent, indicating that Skp can probably bury the surface-exposed tryptophan residues in these OMPs more efficiently than Spy.

To further examine whether the interactions between Spy and OMPs can confer Spy with aggregation prevention activities, we conducted *in vitro* aggregation assays by monitoring the light scattering signals of each OMP under refolding conditions in the presence or absence of different concentrations of Spy and Skp. We found that Spy could prevent all the OMPs, except for OmpT, from aggregation (Fig. 5D), providing direct evidence of Spy’s function in chaperoning OMPs. For Skp, it could strongly inhibit all the OMPs from aggregation (Fig. 5D). Although the inhibition activities of Spy on OMPs were generally lower than Skp, Spy was nearly as efficient as Skp in protecting the 18-β-stranded LamB and the 16-β-stranded OmpC from aggregation (Fig. 5D).

### The structural basis of Spy binding with OMPs.

**FIG 6:**
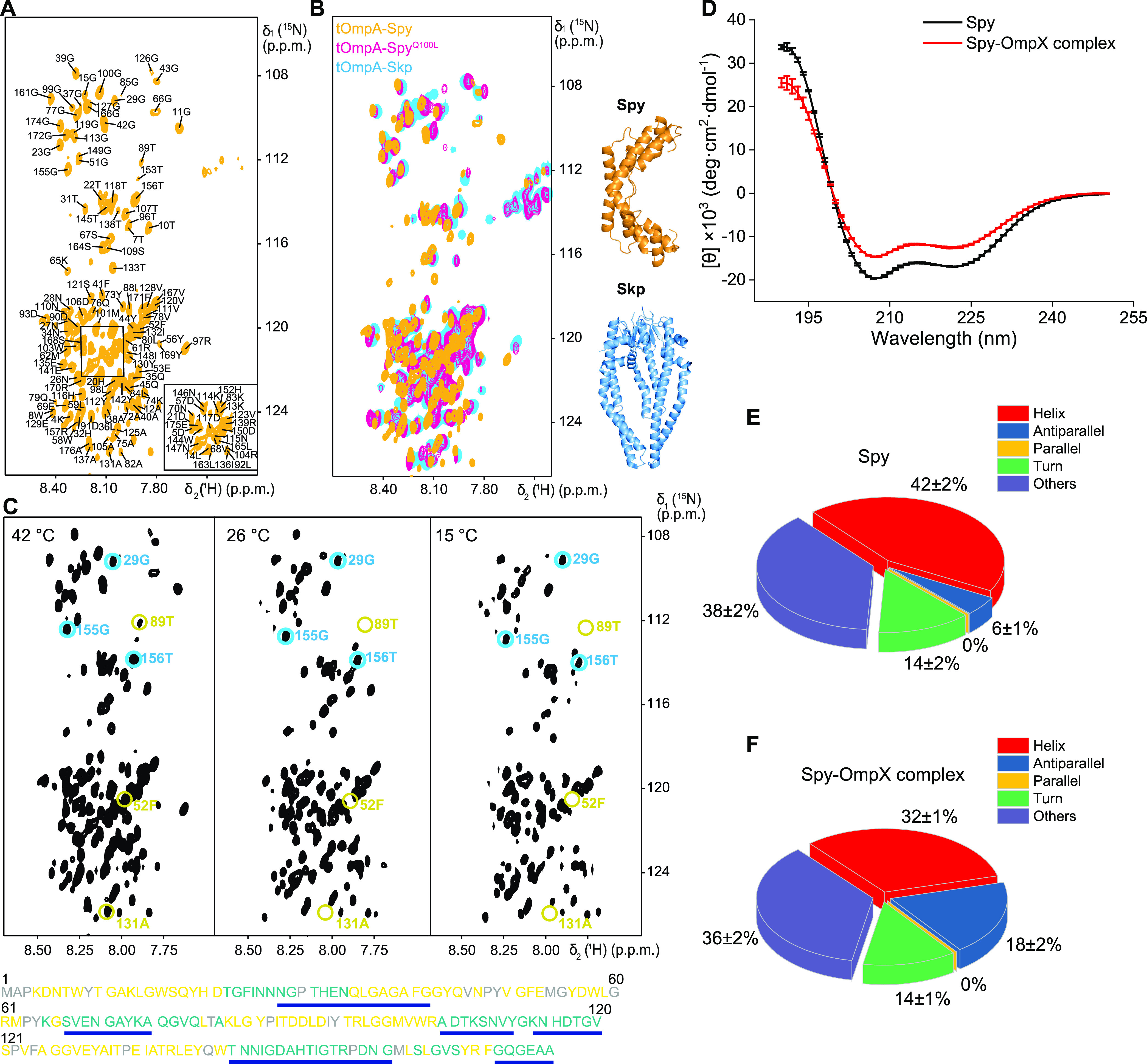
NMR-spectroscopy-based analysis of Spy-bound OMP conformations. (A) Two-dimensional (2D) [^15^N-^1^H]-TROSY fingerprint spectrum of [*U*-^2^H, ^15^N] tOmpA bound to unlabeled Spy. The sequence-specific resonance assignments of the backbone amide groups are indicated. (B) Overlapping of 2D [^15^N-^1^H]-TROSY spectra of [*U*-^2^H, ^15^N] tOmpA bound to unlabeled Spy (orange), Spy^Q100L^ (pink). (C) 2D [^15^N-^1^H]-TROSY spectra of [*U*-^2^H, ^15^N] tOmpA in complex with unlabeled Spy at the temperatures of 42°C, 26°C, and 15°C. Examples for amide resonances with (light yellow) and without (light blue) line broadening in this temperature range are indicated. The amino acid sequence of tOmpA is listed below. Residues with observable and unobservable amide resonances at 15°C are colored in cyan and light yellow, respectively, whereas the unassigned residues are colored in gray. The most hydrophilic peptide segments are highlighted with blue bars. (D) The circular dichroism spectra of Spy (black) and Spy in complex with OmpX (red). Data are shown as mean ± SD (*n* = 3 biological replicates). (E and F) The secondary structure contents of Spy (E) and Spy in complex with OmpX (F) were estimated on the web server BESTSEL (56, 57). Data are shown as mean ± SD (*n* = 3 biological replicates).

To investigate the molecular basis of Spy’s interaction with OMPs, we next characterized the interactions between Spy and OMPs by using nuclear magnetic resonance (NMR) spectroscopy. Specifically, we characterized the complexes of [*U*-^2^H,^15^N]-labeled tOmpA and OmpX with unlabeled Spy using two-dimensional (2D) [^15^N,^1^H] transverse relaxation optimized spectroscopy (TROSY) NMR spectra. The spectrum of tOmpA with Spy showed narrow chemical shift dispersions of the backbone amide protons, reflecting a high mobility of the polypeptide chain as with intrinsically disordered polypeptide chains or chemically denatured proteins (Fig. 6A). This result implied that Spy bound unfolded tOmpA.

We then compared the spectra of tOmpA in complex with Spy, Spy^Q100L^, and Skp. Remarkably, the 2D [^15^N,^1^H]-TROSY NMR spectra of tOmpA bound to the Spy^Q100L^ variant showed 165 resonance peaks and that of tOmpA bound to Skp showed 162 resonance peaks, out of which 140 were overlapping (Fig. 6B). Due to the strong dependence of the NMR chemical shift on protein conformation, the high degree of consistency between the resonance peaks for Spy^Q100L^- and Skp-bound tOmpA indicated that tOmpA retained a similar fluid-globule conformation (8) upon interactions with both chaperones. In contrast, the 2D [^15^N,^1^H]-TROSY spectrum of tOmpA in complex with Spy showed only 83 out of 162 overlapped peaks compared to the tOmpA-Skp spectrum (Fig. 6B), suggesting different conformational ensembles of tOmpA in complex with Spy.

Moreover, temperature-dependent experiments gave us further insights into the dynamic properties of the OMP substrates. Comparing the 2D [^15^N,^1^H]-TROSY NMR spectra of Spy-bound tOmpA recorded at 42°C, 26°C, and 15°C, we observed line broadening in the local hydrophobic segments, with a concomitant signal amplitude decrease below the experimental detection limit (Fig. 6C). For these residues, the local interconversion rate constants moved into the intermediate exchange regime at 15°C, whereas the most hydrophilic polypeptide segments remained in the fast exchange regime at this temperature (Fig. 6C). These temperature and sequential dependent conformational exchanges of OMP within the chaperone pointed to a multitude of local dynamics.

The NMR spectrum of Spy-bound OmpX showed that most peaks were distributed in a narrow range between 8.0 to 8.5 ppm in the ^1^H dimension, revealing that OmpX was also mainly in an unfolded state when bound to Spy (Fig. S3A). However, we also observed a few unexpected peaks in the range around 9 ppm in the ^1^H dimension (Fig. S3A). The dynamic nature of the bound OmpX broadened the NMR peaks lines and precluded the backbone assignment and residual specific analysis of the intermediate state structure. Nevertheless, to gain insight into the secondary structure of OmpX in the complex, we applied circular dichroism (CD) spectroscopy. Assessment of secondary structure contents from CD spectra of Spy-OmpX complex yielded 12% more contents of β-strand secondary structure elements than that of apo-Spy in the same concentration and buffer conditions (Fig. 6D to F). Given that only marginal difference between ^1^H-^15^N heteronuclear single quantum coherence (HSQC) spectra of Spy in the absence and presence of OmpX was observed (Fig. S3B), we could exclude significant conformational changes of Spy, allowing us to ascribe the increased β-strand secondary structure elements to OmpX. Moreover, based on the backbone chemical shift perturbations of Spy, we identified and mapped the interaction sites of OmpX on Spy’s crystal structure (PDB identifier 3O39), which spanned the concave surface of Spy (Fig. S3C and D). For comparison, we also measured the 2D [^15^N,^1^H]-TROSY spectrum of OmpX in the presence of Spy^Q100L^. All of the peaks were distributed between 8.0 to 8.5 ppm (Fig. S3A), which was the same as the published data of Skp-bound OmpX (8). This result indicates that Spy^Q100L^ retains OmpX mainly in the unfolded state, which is consistent with the previously reported finding that Spy^Q100L^ prevents Fyn SH3 from folding more effectively than WT Spy (20). Together, these data suggest that wild-type Spy holds OmpX in predominately unfolded states with partially folded β-strand conformation, providing an alternative chaperone mechanism.

10.1128/mBio.02130-21.3 FIG S3 Interactions between OMPs and chaperone Spy, Spy^Q100L^, and Skp. (A) Overlapping of 2D [^15^N-^1^H]-TROSY spectra of [*U*-^2^H, ^15^N] OmpX bound to unlabeled Spy (green) and [*U*-^2^H, ^15^N] OmpX bound to Spy^Q100L^ (pale blue). (B) Overlapping of 2D [^15^N-^1^H]-HSQC spectra of [*U*-^2^H, ^15^N] Spy (cyan) and [*U*-^2^H, ^15^N] Spy in complex with unlabeled OmpX (purple). (C) Chemical shift perturbations (CSPs) of amide moieties of [*U*-^2^H, ^15^N] Spy in complex with OmpX, plotted against the Spy amino acid residue number. (D) Structural representation of the CSPs mapped on the Spy crystal structure (PDB identifier 3O39). A gray-to-orange color scale is applied according to the magnitude of the CSPs for the interaction with OmpX. The brightest orange indicates the largest CSP. Download FIG S3, PDF file, 1.5 MB. Copyright © 2021 He et al. 2021 He et al. https://creativecommons.org/licenses/by/4.0/ This content is distributed under the terms of the Creative Commons Attribution 4.0 International license.

## DISCUSSION

Our *in vivo* and *in vitro* results suggest that Spy, a chaperone proposed to be promiscuous toward many periplasmic proteins (37), also contributes to maintaining the proteostasis of certain OMPs. Our observation that overexpression of Spy leading to the recovery of OmpX, OmpC, and OmpA levels in a *skp fkpA* double deletion mutant supports the idea that Spy may be functionally redundant to Skp and FkpA toward certain OMP targets. Based on the above observation, as well as the physical interactions between Spy and certain OMPs detected *in vitro*, we assume that Spy can bind to unfolded (or largely unfolded) OMPs *in vivo*, preventing them from aggregating and being subjected to degradation. However, unlike FkpA—whose chaperone activity was drastically enhanced by the heat shock stress condition (10)—we did not observe a butanol-dependent increase in the activity of Spy; instead, we saw a 30% loss of activity for Spy and a 20% loss of activity for Skp after 30 min of 1% butanol stress treatment (see Fig. S4 in the supplemental material). These results support that Spy acts primarily through stress-induced overexpression, a finding consistent with the known fact that Spy accounts for nearly 20% of the total periplasmic protein content in butanol-stressed cells (15).

10.1128/mBio.02130-21.4 FIG S4 Spy and Skp exhibit decreased antiaggregation activities towards OmpX in the presence of 1% butanol. (A) The antiaggregation activities of Spy or Skp towards OmpX were monitored in the presence and absence of 1% butanol. Spy (30 μM) or Skp (4.5 μM) were incubated for 30 min in 40 mM HEPES-150 mM NaCl (pH 7.5) buffer supplied with or without 1% butanol (vol/vol), and then OmpX (150 μM in 8 M urea) was added to a final concentration of 1.5 μM. OmpX aggregation was monitored by the light scattering signals at 380 nm. The antiaggregation activities of Spy and Skp were expressed as the proportion of OmpX aggregation, while OmpX aggregation in the absence of any chaperone in the presence or absence of 1% butanol represented complete aggregation. (B) Both Spy and Skp showed decreased antiaggregation activities towards OmpX when treated with 1% butanol. Mean ± SD; *n* = 3; individual data points are shown. Unpaired two-tailed Student’s *t* test; **, *P* < 0.01; ***, *P* < 0.001. Download FIG S4, PDF file, 0.5 MB. Copyright © 2021 He et al. 2021 He et al. https://creativecommons.org/licenses/by/4.0/ This content is distributed under the terms of the Creative Commons Attribution 4.0 International license.

Our NMR experiments demonstrated that Spy-bound OMPs were maintained in a predominantly unfolded state. Similar trends were detected for Skp- and SurA-bound OMPs (38, 39), indicating some similarities among these ATP-independent chaperones when in complex with OMPs. However, there are differences in the detailed substrate-binding mechanisms between these chaperones. Upon forming chaperone-tOmpA complex, Skp compacts unfolded tOmpA in a fluid-globule ensemble, while SurA expands unfolded tOmpA, possibly to more efficiently deliver OMPs to the BAM complex (8, 40). Although Spy’s exact mode of action is still unknown, we propose that Spy may enable its client OMPs to adopt compact states, more similarly to Skp than SurA. Two lines of evidence can support this supposition. Spy has already been shown to compact the water-soluble substrate Im7 (17, 20, 41), and the resonance positions of tOmpA when bound to Skp and Spy are highly coincident. For OmpX, a partially folded β-strand secondary structure was detected while it was bound to Spy, suggesting that Spy has the potential to support OmpX partially folding. This mechanism is different from that of Skp and SurA, in which the bound OMPs with these chaperones display totally unfolded ensembles (8, 38, 39). The previously reported folding-while-bound mechanism of Spy may account for the local structure formation of OmpX; that is, both weak interactions between Spy and OMPs and the local conformational flexibility of Spy may enable specific regions of OmpX to search for a defined local structure upon Spy binding (19, 20). This remarkable property of Spy may render the clients competent for the subsequent insertion into the OM.

Understanding how periplasmic chaperones bind and release their client proteins, without any obvious source of ATP, represents a fundamental question for studies investigating chaperone actions in the periplasm. For SurA, its interdomain dynamics enable it to adopt “open” and “closed” conformations in solution. The open state promotes client binding, while the closed state blocks client’s entrance (42), suggesting that SurA may adjust client binding and release through dynamic conformational changes. Skp adopts a more stringent regulatory mechanism by combining “disorder-to-order transition” and “oligomer assembly” to couple its chaperone cycle (43). It is suggested that only Skp trimers are able to bind OMP substrates and that about half of the Skp protein population exists in an inactive and intrinsically disordered monomer state under physiological conditions (43). OMP binding can shift this equilibrium toward the formation of the active trimer state (43), and thus the binding of substrates is a synergistic process. The mechanism of how Skp releases OMP substrates is still not understood; however, a decreased trimer-monomer ratio of Skp was proposed to account for enhanced substrate release kinetics. For Spy, Horowitz et al. and we observed neither dramatic conformation changes nor dimer-monomer transitions during its chaperone cycle for water-soluble client proteins (17, 44). Considering our observations that binding with Spy supports local structure formation for OmpX, it is reasonable to speculate that self-folding of the OMPs may provide (at least partially) the driving force for client release.

Currently, it remains unclear whether Spy hands over these OMP clients to other chaperones like SurA and Skp or whether it escorts them directly to the Bam complex or the OM. It was previously proposed that the positively charged residues at the tips of the α-helical tentacles of Skp may mediate electrostatic interactions between Skp and the negatively charged OM, allowing Skp to directly hand over OMPs for membrane insertion (45–47). Spy, with an overall positively charged and concave substrate-binding surface, may also engage with the OM in a similar manner as Skp. Further investigations about whether Spy is capable of accelerating the folding rates of OMPs in reconstituted proteoliposomes (48, 49) and whether SurA, Skp, or BamA affect this process will help answer these questions.

In conclusion, our study has shown that Spy is able to maintain certain OMP homeostasis in the absence of other major OMP chaperones and illustrates an alternative mode of action of periplasmic chaperones toward OMP clients. Understanding the various aspects of OMP biogenesis pathways will facilitate the design of inhibitors for specific chaperone-substrate interactions, which ultimately provides a novel approach to combat life-threatening pathogenic bacteria.

## MATERIALS AND METHODS

### Strains and plasmids.

The strains and plasmids used in this study are summarized in Table 1. Gene knockout and site-directed point mutations on the E. coli chromosome were performed with the phage λ Red recombinase system as described previously (50, 51). Plasmids were constructed by the QuikChange site-directed mutagenesis method or by the overlap extension PCR cloning method (52). All strains were cultivated in LB medium supplemented with 50 μg/ml streptomycin, 50 μg/ml ampicillin, or 25 or 100 μg/ml kanamycin when necessary, under aerobic conditions at 37°C.

### Spot titer experiments.

Overnight cell cultures (optical density at 600 nm [OD_600_] = 1) were spun down, resuspended with 1 ml of 0.17 M NaCl, and serially diluted to yield cell titers with an OD_600_ of 10^0^ to 10^−5^. Next, 2 μl of each cell titer was spotted on LB plates or LB plates containing 2.2 mM EDTA, or 75 μg/ml clindamycin, or 6.5% (vol/vol) ethanol, or various concentrations of novobiocin, and then grown overnight at 37°C.

### Polymyxin B susceptibility test.

WT or Δ*spy* strain overnight cell cultures were 1:100 diluted into 50 ml LB medium and cultured at 37°C. When the OD_600_ reached 1, polymyxin B was added to a final concentration of 100 μg/ml. The OD_600_ of each strain was recorded at each time point.

### 1-*N*-phenylnaphthylamine uptake assay.

WT or Δ*spy* strain cells were cultured at 37°C to an OD_600_ of 0.8 before adding butanol to a final concentration of 1% (vol/vol). At each time point, 1 OD_600_ of cells was spun down and resuspended with 1 ml of a 5 mM HEPES (pH 7.2) buffer. Then, 0.5 ml of 40 μM NPN stock and 0.5 ml of the 5 mM HEPES (pH 7.2) buffer were added into the cell suspension. The NPN fluorescence was monitored at 25°C with the excitation and emission wavelength at 340 nm and 407 nm, respectively, using a Lumina fluorescence spectrometer supplied with a Peltier temperature controller (Thermo Fisher Scientific). The NPN uptake factors were calculated as follows:

$$\text{NPN uptake factor}=(F_{\text{obs}}-F_{\text{cell}})/(F_{\text{NPN}}-F_{\text{buffer}})$$

where *F*_obs_ is the measured fluorescence of NPN with cells, *F*_cell_ is the fluorescence of cells alone, *F*_NPN_ is the fluorescence of NPN in the absence of cells, and *F*_buffer_ is the fluorescence of buffer only.

### LPS extraction.

Cells were cultivated and collected as described in “Sample preparation for proteomics” in Text S1. The extraction procedure was conducted as previously described (53), with the following modifications. After cell lysis, benzonase with a final concentration of 6.25 U/ml was used to degrade the DNA and RNA contaminants. Finally, a 10 μl extraction of each strain was separated with a 16% tricine gel, following by standard silver staining.

### Western blot analysis.

Overnight cell cultures were inoculated at a 1:100 dilution-fold into 50 ml fresh LB, and cells were cultivated at 37°C to reach an OD_600_ of ∼0.7. Then, 15 ml of cells was spun down and resuspended with a 50 mM Tris-HCl (pH 6.8) buffer to an OD_600_ of 20. Protease inhibitor (100×, DI111-01; TransGen), 0.15 mg/ml lysozyme, and 2.5 U/ml benzonace were added into the cell suspension, followed by 6 freeze-thaw cycles. Next, 2× reducing SDS/PAGE sample buffer (4% β-mercaptoethanol [β-ME], 4% SDS, 20% glycerol and 100 mM Tris-HCl [pH 6.8]) was added to 200 μl cell lysate, and samples were divided into two aliquots. One was boiled for 10 min for denaturing SDS/PAGE separation to blot for the total protein level of each OMP, and the other was not boiled for seminative SDS/PAGE separation to detect the folded protein levels of each OMP. For detecting mature LptD, 2× nonreducing SDS/PAGE sample buffer (4% SDS, 20% glycerol, and 100 mM Tris-HCl [pH 6.8]) was added and the cell lysate was boiled before SDS/PAGE. For effectively transferring the folded OMPs that were wrapped by lipopolysaccharides (10), the seminative SDS/PAGE gels were heated by steaming for 10 min before standard wet transfer to 0.2 μM polyvinylidene difluoride (PVDF) membranes. All primary antibodies were used at a 1:1,000 dilution except for LptD (1:800), LamB (1:5,000), and GAPDH (1:6,000). IRDye 800CW goat anti-rabbit IgG secondary antibody (LI-COR, catalog no. 926-32211) was used at the 1:10,000 dilution.

### Protein purification.

Spy and Skp were purified as described previously (16), except for the following modifications. The 6×His-SUMO-tagged Spy and Skp were first eluted, and then the 6×His-SUMO tag was removed and the buffer was changed to the cation exchange buffer A (20 mM HEPES and 0.5 mM EDTA [pH 7.5]).

For OMPs used in the intrinsic tryptophan fluorescence assay and aggregation assay, a 6×His tag was fused at the N terminus of the mature sequence of each OMP, and the purification process was performed according to Lyu et al. (54).

For OMPs used for NMR spectroscopy, no affinity tags were used, and the purification was performed as previously described with minor adaptions (55). Briefly, cells were cultured in M9 medium supplied with heavy water (D_2_O) and 1 g/liter ^15^NH_4_Cl at 37°C to an OD_600_ of ∼0.7. Then, 0.2 mM isopropyl-β-d-thiogalactopyranoside (IPTG) was added to induce protein expression. After growing at 37°C for 12 h, 1-liter cultures were centrifuged, and the cells were resuspended in 40 ml TE buffer (20 mM Tris-HCl and 5 mM EDTA [pH 8.5]) supplied with protease inhibitors. Cells were lysed by a high-pressure homogenizer, and the pellet was separated by centrifugation at 10,000 × *g* and 4°C for 1 h. Then, the pellet was washed sequentially with 40 ml TET buffer (20 mM Tris-HCl, 5 mM EDTA, and 2% Triton [vol/vol] [pH 8.5]) and TE buffer at 37°C for 1 h. After each wash, the cell pellet was separated by centrifugation at 5,000 × *g* and 4°C for 30 min. Then, 12 ml TEU buffer (20 mM Tris-HCl, 5 mM EDTA, and 8 M urea [pH 8.5]) was added to solubilize the pellet at 37°C for 1.5 h. The insoluble contaminants were removed by centrifugation at 21,000 × *g* and 25°C, and the supernatant was subject to further purification by ion exchange chromatography. The final elution fractions containing tOmpA or OmpX were concentrated and stored at −80°C before usage.

### Intrinsic tryptophan fluorescence spectroscopy.

The interactions between OMPs and chaperones were detected by intrinsic tryptophan fluorescence spectroscopy using a Lumina fluorescence spectrometer supplied with a Peltier temperature controller (Thermo Fisher Scientific). OMPs were dialyzed into buffer A, containing 40 mM HEPES, 150 mM NaCl, and 8 M urea [pH = 7.5], whereas Skp and Spy were dialyzed into buffer B, composed of 40 mM HEPES, 150 mM NaCl, and 0.24 M urea [pH = 7.5] before fluorescence measurement. To initiate the assay, OMPs were diluted with calculated ratios of buffer A and buffer C (40 mM HEPES, and 150 mM NaCl [pH = 7.5]) to achieve a final concentration of 0.4 μM OMP protein and 0.24 M urea in a buffer containing 40 mM HEPES and 150 mM NaCl (pH = 7.5). Then concentrated Skp and Spy were titrated into the solution to obtain the appropriate molar ratios. The tryptophan fluorescence of OMPs in the absence and presence of increasing molar ratios of Spy and Skp were excited at a wavelength of 295 nm and monitored in the range of 305 nm to 400 nm at 25°C.

### OMP aggregation assay.

The aggregation of OMPs at 25°C was monitored by light scattering at 360 nm using a Lumina fluorescence spectrometer (Thermo Fisher Scientific). Spy and Skp were first diluted to appropriate molar ratios toward OMPs in a buffer containing 50 mM NaP and 100 mM NaCl (pH = 7.0). Then, concentrated OMPs in the 50 mM NaP,100 mM NaCl, and 8 M urea (pH = 7.0) buffer was diluted 100-fold into the Spy or Skp-containing solution to reach a final concentration of 1.5 μM and a total volume of 1 ml. After stirring for 30 s, the cuvette containing the mixture was transferred into the spectrometer for data recording.

### Assembly of chaperone-OMP complexes.

Chaperone-OMP complexes were prepared according to a published protocol (39). Briefly, an excess of urea-denatured OMPs was titrated into chaperones in the assembly buffer (25 mM HEPES, 150 mM NaCl, and 1 mM dithiothreitol [DTT] [pH 7.5]) in a dropwise fashion under continuous stirring at room temperature. To ensure saturation of chaperones, OMPs were added until the precipitation was observable. The solution was then stirred for another 1 h. After centrifugation at 12,000 × *g* for 30 min, the supernatant was concentrated by ultrafiltration, and assembly buffer was exchanged to NMR buffer (25 mM morpholineethanesulfonic acid [MES] and 50 mM NaCl [pH 6.5]).

### NMR spectroscopy.

All NMR experiments for OMP-chaperone complexes were performed in NMR buffer (25 mM MES and 50 mM NaCl [pH 6.5]). tOmpA-Skp and tOmpA-Spy^Q100L^ complexes were recorded at 315 K on Bruker Ascend II 700-MHz spectrometers equipped with a cryogenically cooled triple-resonance probe. The OmpX-Spy complex was recorded at 315 K on Avance 850-MHz spectrometers with a cryogenically cooled triple-resonance probe. The interscan delay was set to 1 s. In the direct dimension, 2,048 complex points were recorded, multiplied with a 75°-shifted sine bell, zero-filled to 4,096 points, and Fourier transformed. In the indirect dimension, 160 complex points were measured, multiplied with a 75°-shifted sine bell, zero-filled to 256 points, and Fourier transformed. Polynomial baseline correction was applied in all dimensions.

### Circular dichroism spectroscopy.

Circular dichroism (CD) spectra were recorded on a Chirascan instrument using a cell of 1-mm path length. Spectral sampling parameters include a scanning rate at 0.5 s/point and a bandwidth of 1 nm, in the far-UV wavelength range of 190 to 250 nm. Each spectrum was obtained from an average of 5 scans. The CD data are shown in terms of the mean residue ellipticity as a function of wavelength. The protein concentrations of 0.192 mg/ml Spy and 0.192 mg/ml Spy in complex with 0.045 mg/ml OmpX were used for CD measurement.
