# Supplementary material for: Chaperone Spy Protects Outer Membrane Proteins from Folding Stress via Dynamic Complex Formation
Source: mBio. 2021 Oct 5;12(5):e02130-21. doi: 10.1128/mBio.02130-21 (PMC8546600; doi:10.1128/mBio.02130-21)
Supplement: TABLE S1 [file mbio.02130-21-st001.docx]

**TABLE S1** Summary of proteins with a decreased abundance in the *spy* containing strains

| Protein | Fold change | p-value | Identified comparison group | Topology Class*^a^* | Description |
| --- | --- | --- | --- | --- | --- |
| RpsU | 2.6 | 0.013 | *∆spy*/WT | cytoplasmic protein | ribosomal small subunit assembly |
| GltX | 3.0 | 0.025 | *∆spy*/L32P | cytoplasmic protein | glutamate--tRNA ligase |
| LpdA | 2.0 | 0.022 | *∆spy*/L32P | peripheral inner membrane protein facing the cytoplasm | dihydroyl dehydrogenase |
| FabF | 4.2  4.3 | 0.013  0.011 | *∆spy*/WT  *∆spy*/L32P | peripheral inner membrane protein facing the cytoplasm | 3-oxoacyl-(acyl-carrier-protein) synthase |
| SuhB | 2.1 | 3.2E-04 | *∆spy*/Q100L | peripheral inner membrane protein facing the cytoplasm | part of the processive rRNA transcription and antitermination complex (rrnTAC) |
| UcpA | 2.2 | 0.013 | *∆spy*/Q100L | cytoplasmic protein | oxidoreductase |
| BtuR | 2.5 | 0.004 | *∆spy*/Q100L | cytoplasmic protein | corrinoid adenosyltransferase |
| HldE | 2.0  2.1 | 0.012  0.007 | *∆spy*/WT  *∆spy*/Q100L | cytoplasmic protein | catalyzes the phosphorylation of D-glycero-D-manno-heptose 7-phosphate |
| YhcH | 11.6*^b^*  (absent in WT) | 1.12E-04 | *∆spy*/WT | cytoplasmic protein | uncharacterized |
| MurC | 12.8*^b^*  (absent in WT) | 0.002 | *∆spy*/WT | peripheral inner membrane protein facing the cytoplasm | cell wall formation |
| SecG | 32.4*^b^*  (absent in L32P) | 5.13E-04 | *∆spy*/L32P | integral inner membrane protein | protein exportation |
| DnaN | 8.9*^b^*  (absent in L32P) | 3.71E-04 | *∆spy*/WT  *∆spy*/L32P | cytoplasmic protein | beta sliding clamp, confers DNA tethering and processivity to DNA polymerases and other proteins |
| YbiT | 22.3*^b^*  (absent in WT and L32P) | 0.003 | *∆spy*/WT  *∆spy*/L32P | peripheral inner membrane protein facing the cytoplasm | ABC transporter, ATP-binding |
| YibL | 28.5*^b^*  (absent in WT and L32P) | 1.38E-05 | *∆spy*/WT  *∆spy*/L32P | cytoplasmic protein | uncharacterized |
| ZntR | 6.8*^b^*  (absent in Q100L) | 2.74E-05 | *∆spy*/Q100L | cytoplasmic protein | HTH-type transcriptional regulator |
| SufS | 9.1*^b^*  (absent in Q100L) | 1.52E-04 | *∆spy*/Q100L | peripheral inner membrane protein facing the cytoplasm | cysteine desulfuration |
| RimJ | 10.3*^b^*  (absent in Q100L) | 2.11E-04 | *∆spy*/Q100L | peripheral inner membrane protein facing the cytoplasm | ribosomal-protein-alanine acetyltransferase |
| BioD2 | 11.8*^b^*  (absent in Q100L) | 2.11E-04 | *∆spy*/Q100L | cytoplasmic protein | ATP-dependent dethiobiotin synthetase |
| DedD | 18.4*^b^*  (absent in Q100L) | 4.20E-04 | *∆spy*/Q100L | integral inner membrane protein | cell division |
| FolD | 25.8*^b^*  (absent in Q100L) | 2.08E-05 | *∆spy*/Q100L | cytoplasmic protein | methylenetetrahydrofolate dehydrogenase; methenyltetrahydrofolate cyclohydrolase |
| Mog | 46.4*^b^*  (absent in WT and Q100L) | 5.67E-04 | ∆spy/WT  *∆spy*/Q100L | cytoplasmic protein | molybdopterin adenylyltransferase |
| RsmE | 6.2*^b^*  (absent in L32P and Q100L) | 0.002 | *∆spy*/L32P  *∆spy*/Q100L | peripheral inner membrane protein facing the cytoplasm | ribosomal RNA small subunit methyltransferase |
| Tdk | 12.4*^b^*  (absent in L32P and Q100L) | 3.54E-06 | *∆spy*/L32P  *∆spy*/Q100L | cytoplasmic protein | thymidine kinase |
| SdhB | 2.0*^b^*  36.7*^b^*  (absent in L32P and Q100L) | 0.021  3.58E-06 | *∆spy*/WT  *∆spy*/L32P  *∆spy*/Q100L | peripheral inner membrane protein facing the cytoplasm | catalysis of fumarate and succinate interconversion |
| ProV | 16.1*^b^*  (absent in WT, L32P, and Q100L) | 8.20E-09 | *∆spy*/WT  *∆spy*/L32P *∆spy*/Q100L | peripheral inner membrane protein facing the cytoplasm | part of the ProU ABC transporter complex involved in glycine betaine and proline betaine uptake |

*^a^*The topology class of each protein was noted according to sub-cellular topology and localization of the Escherichia coli polypeptides (STEPdb).

*^b^*The missing LFQ values were imputed using Deterministic Minimal Imputation as described in the supplemental materials and methods.
