# Supplementary material for: Chaperone Spy Protects Outer Membrane Proteins from Folding Stress via Dynamic Complex Formation
Source: mBio. 2021 Oct 5;12(5):e02130-21. doi: 10.1128/mBio.02130-21 (PMC8546600; doi:10.1128/mBio.02130-21)
Supplement: TEXT S1 [file mbio.02130-21-s0001.docx]

**SUPPLEMENTAL MATERIALS AND METHODS**

**Sample preparation for proteomics**

Overnight cultures with 3 biological replicates for each strain were 1:100 diluted into LB broth, shaking at 220 rpm at 37 °C to reach an optical density of OD_600_ ~ 0.4. Then, butanol was added to each culture to a final concentration of 1 % (v/v). After 1.5 h continue shaking, cells were harvested by centrifugation at 3,000 ×g for 20 min at 4 °C. Cell envelope proteins were obtained by a modified osmotic shock procedure (TSE) as described in (1) followed by acetone precipitation. Protein pellets were dissolved in 50 mM Tris-HCl, pH 8.5 with 8 M urea and protease inhibitors and adjust to a concentration of 3 mg/ml. DTT was added to the mixture to a final concentration of 10 mM to reduce the proteins. After incubation at 37 °C for 2.5 h, alkylation was performed by addition of 50 mM iodoacetic acid and incubation at 25 °C for 40 min in the dark. Then, 4 volumes of 100 mM ammonium bicarbonate were added to the reaction to reduce urea concentration. Trypsin digestion of the proteins (trypsin/proteins mass ratio 1:25) was performed in the above mixture at 37 °C for 24 h with gentle rocking. The resulting peptides were separated from the undigested proteins by centrifugation in a 10 kDa-cutoff concentrator (Millipore) and the flow through was collected. Protein concentration for the above procedure was determined by Bradford assay (Pierce).

**Nanoflow LC-MS/MS**

1 μg of peptide digests dissolved in 0.1 % (v/v) formic acid were loaded onto an in-house packed 20 cm capillary column with 3 µm Reprosil-Pur C18 beads (Dr. Maisch, Ammerbuch, Germany) using an EASY-nLC 1000 system (Thermo Scientific, San Jose, USA). Mobile phase buffers for the LC separation consisted of 0.1 % (v/v) formic acid in water (buffer A) and 0.1 % (v/v) formic acid in acetonitrile (buffer B). The peptides were eluted during a 2 h gradient and directly sprayed into the mass spectrometer. The flow rate started at 350 nl/min, and the detailed LC gradient was performed as follows: 0 % B for 11 min, 8-30 % B in 105 min, and 30-90 % B in 4 min. MS data were acquired using a data-dependent top-20 method on Q Exactive (Thermo Scientific, Bremen, Germany). Spray voltage was set to 2 kV, S-lens RF level at 60, and capillary temperature at 275 °C. Full scan resolutions were set to 60,000 at m/z 200 and AGC was 3×106 with a maximum fill time of 20 ms. Mass range of full mass was set to 350-1500. MS2 scan resolutions were set to 15,000 at m/z 200 and AGC was 5×104 with a maximum fill time of 45 ms. Isolation width was set at 1.6 Th. A fixed first mass of 110 was used. Normalized collision energy was set at 27. Peptide match was set to “preferred” and isotope exclusion was on. Precursor ions with single, un-assigned, charge states were removed from fragmentation selection (2-4).

**Protein identification and statistical analysis**

For protein identification and quantification, we used MaxQuant software (version 1.5.1.12) and its integrated search engine Andromeda for matching the MS/MS spectra with proteins from UniProt database of the *E.coli* K12 proteome (version 201504; 4269 entries) combined with 262 common contaminants (5, 6). The following digestion parameters were used: enzyme specificity was set to trypsin, allowing maximum of two miscleavages. For protein identification, only peptides above five amino acids and a maximum mass of 10,000 Da, were considered. Cysteine carbamidomethylation was set as a fixed modification, while methionine oxidation were set as variable modifications. A false discovery rate of 0.01 was applied at the protein and peptide levels. Only entries that have been identified with at least two unique peptides and quantified in at least three biological replicates were considered for statistical data analysis.

Quantification and comparison of the protein abundance were done using label-free quantification (LFQ) derived by the MaxQuant software, applying the default parameters. Only proteins identified in all three biological replicates in at least one protein group were considered. To obtain the volcano plot and fold change we replaced missing LFQ values with minimal values smaller than the observed LFQ values in the entire dataset, following the Deterministic Minimum Imputation method (7). To ensure that the imputation didn’t affect the statistical comparison of other proteins, we compared the distribution of all the LFQ values in the dataset before and after the imputation. Moreover, the fold change of the significantly different proteins with valid LFQ values was not affected by the imputation. The two-tailed unpaired student’s t-test was used to identify significantly different proteins, which had p-value <0.05, and fold change more than 2.

**REFERENCES**

1. Quan S, Hiniker A, Collet JF, Bardwell JC. 2013. Isolation of bacteria envelope proteins. Methods Mol Biol 966:359-66.

2. Kelstrup CD, Jersie-Christensen RR, Batth TS, Arrey TN, Kuehn A, Kellmann M, Olsen JV. 2014. Rapid and deep proteomes by faster sequencing on a benchtop quadrupole ultra-high-field Orbitrap mass spectrometer. J Proteome Res 13:6187-95.

3. Kelstrup CD, Young C, Lavallee R, Nielsen ML, Olsen JV. 2012. Optimized fast and sensitive acquisition methods for shotgun proteomics on a quadrupole orbitrap mass spectrometer. J Proteome Res 11:3487-97.

4. Scheltema RA, Hauschild JP, Lange O, Hornburg D, Denisov E, Damoc E, Kuehn A, Makarov A, Mann M. 2014. The Q Exactive HF, a Benchtop mass spectrometer with a pre-filter, high-performance quadrupole and an ultra-high-field Orbitrap analyzer. Mol Cell Proteomics 13:3698-708.

5. Cox J, Mann M. 2008. MaxQuant enables high peptide identification rates, individualized p.p.b.-range mass accuracies and proteome-wide protein quantification. Nat Biotechnol 26:1367-72.

6. Cox J, Neuhauser N, Michalski A, Scheltema RA, Olsen JV, Mann M. 2011. Andromeda: a peptide search engine integrated into the MaxQuant environment. J Proteome Res 10:1794-805.

7. Lazar C, Gatto L, Ferro M, Bruley C, Burger T. 2016. Accounting for the Multiple Natures of Missing Values in Label-Free Quantitative Proteomics Data Sets to Compare Imputation Strategies. J Proteome Res 15:1116-25.
