# Supplementary material for: Chaperone Spy Protects Outer Membrane Proteins from Folding Stress via Dynamic Complex Formation
Source: mBio. 2021 Oct 5;12(5):e02130-21. doi: 10.1128/mBio.02130-21 (PMC8546600; doi:10.1128/mBio.02130-21)
Supplement: FIG S1 [file mbio.02130-21-sf001.pdf]

FIG S1

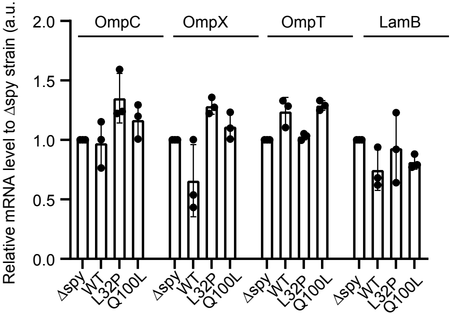

FIG S1 Quantitative RT-PCR analyses of OmpC, OmpX, OmpT, and LamB mRNA levels in *spy*-containing strains relative to *Δspy* strain. The mRNA samples were extracted from mid-log phase cells treated with 1% (v/v) butanol for 1.5 h. The maximal values for the mRNA levels of the 4 OMPs in *spy*-containing strains (WT, L32P, and Q100L) are less than 1.5-fold of the mRNA levels in *Δspy*, which is insufficient to entirely explain the increase of OMP protein levels in *spy*-containing strains (> 2.1 fold). All results are expressed as the Mean ± SD, individual data points derived from 3 biological samples, each with 3 technical repeats.
