# Supplementary material for: Chaperone Spy Protects Outer Membrane Proteins from Folding Stress via Dynamic Complex Formation
Source: mBio. 2021 Oct 5;12(5):e02130-21. doi: 10.1128/mBio.02130-21 (PMC8546600; doi:10.1128/mBio.02130-21)
Supplement: TABLE S2 [file mbio.02130-21-st002.docx]

**TABLE S2** Summary of proteins with a significantly different abundance in the L32P and Q100L strains compared with the WT strain

| Protein more abundant in the L32P and Q100L strains | | | | | |
| --- | --- | --- | --- | --- | --- |
| Protein | Fold change | p-value | Identified comparison group | Topology Class*^a^* | Description |
| OmpC | 13.4/  8.1 | 0.003/  0.005 | L32P /WT  Q100L/ WT | outer membrane β-barrel protein | outer membrane pores allowing for small molecular diffusion |
| RecA | 2.3 | 0.009 | L32P /WT | peripheral inner membrane protein facing the cytoplasm | homologous recombination |
| SrlD | 3.8 | 0.032 | L32P /WT | cytoplasmic protein | sorbitol-6-phosphate 2-dehydrogenase |
| DacA | 5.1 | 0.037 | Q100L/ WT | periplasmic protein | carboxypeptidase involved in peptidoglycan biosynthesis |
| EntC | 2.3 | 0.038 | Q100L/ WT | cytoplasmic protein | biosynthesis of the siderophore enterobactin |
|  |  |  |  |  |  |
| Protein more abundant in the WT strain | | | | | |
| Protein | Fold change | p-value | Identified comparison group | Topology Class^a^ | Description |
| AdhE | 2.4 | 0.029 | WT/L32P | peripheral inner membrane protein facing the cytoplasm | aldehyde-alcohol dehydrogenase |
| NuoG | 6.7 | 0.043 | WT/L32P | peripheral inner membrane protein facing the cytoplasm | NADH-quinone oxidoreductase subunit G |
| RplL | 2.4 | 0.036 | WT/L32P | cytoplasmic protein | 50S ribosomal protein |
| RpsS | 3.6 | 0.009 | WT/L32P | cytoplasmic protein | 30S ribosomal protein |
| YccX | 2.2 | 0.036 | WT/L32P | cytoplasmic protein | acylphosphatase |
| YcbX | 2.4 | 0.011 | WT/Q100L | cytoplasmic protein | uncharacterized |

*^a^*The topology class of each protein was noted according to sub-cellular topology and localization of the Escherichia coli polypeptides (STEPdb).
