# Supplementary material for: Chaperone Spy Protects Outer Membrane Proteins from Folding Stress via Dynamic Complex Formation
Source: mBio. 2021 Oct 5;12(5):e02130-21. doi: 10.1128/mBio.02130-21 (PMC8546600; doi:10.1128/mBio.02130-21)
Supplement: FIG S3 [file mbio.02130-21-sf003.pdf]

FIG S3

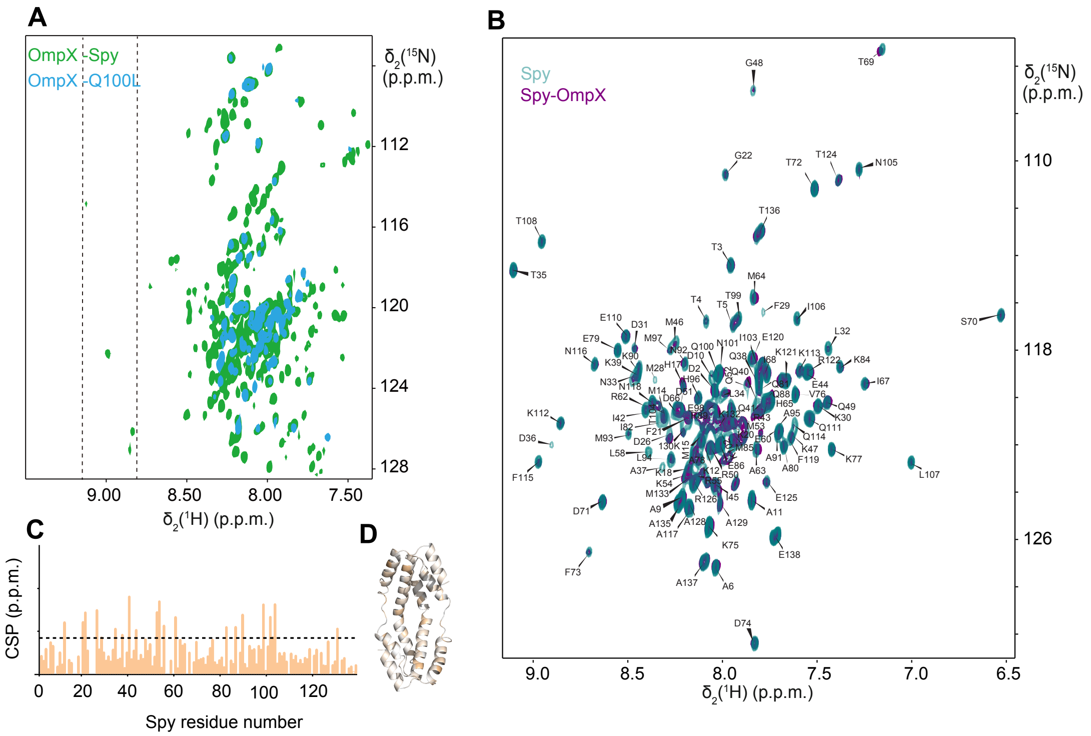

FIG S3 Interactions between OMPs and chaperone Spy, Spy^{Q100L} and Skp. (A) Overlapping of 2D [^{15}N-^{1}H]-TROSY spectra of [*U*-^{2}H, ^{15}N] OmpX bound to unlabeled Spy (green) and [*U*-^{2}H, ^{15}N] OmpX bound to Spy^{Q100L} (pale blue). (B) Overlapping of 2D [^{15}N-^{1}H]-HSQC spectra of [*U*-^{2}H, ^{15}N] Spy (cyan) and [*U*-^{2}H, ^{15}N] Spy in complex with unlabeled OmpX (purple). (C) Chemical shift perturbations (CSPs) of amide moieties of [*U*-^{2}H, ^{15}N] Spy in complex with OmpX, plotted against the Spy amino acid residue number. (D) Structural representation of the CSPs mapped on the Spy crystal structure [ref. PDB: 3O39]. A gray-to-orange color scale is applied according to the magnitude of the CSPs for the interaction with OmpX. The brightest orange indicates the largest CSP.
