# Supplementary material for: Chaperone Spy Protects Outer Membrane Proteins from Folding Stress via Dynamic Complex Formation
Source: mBio. 2021 Oct 5;12(5):e02130-21. doi: 10.1128/mBio.02130-21 (PMC8546600; doi:10.1128/mBio.02130-21)
Supplement: FIG S4 [file mbio.02130-21-sf004.pdf]

FIG S4

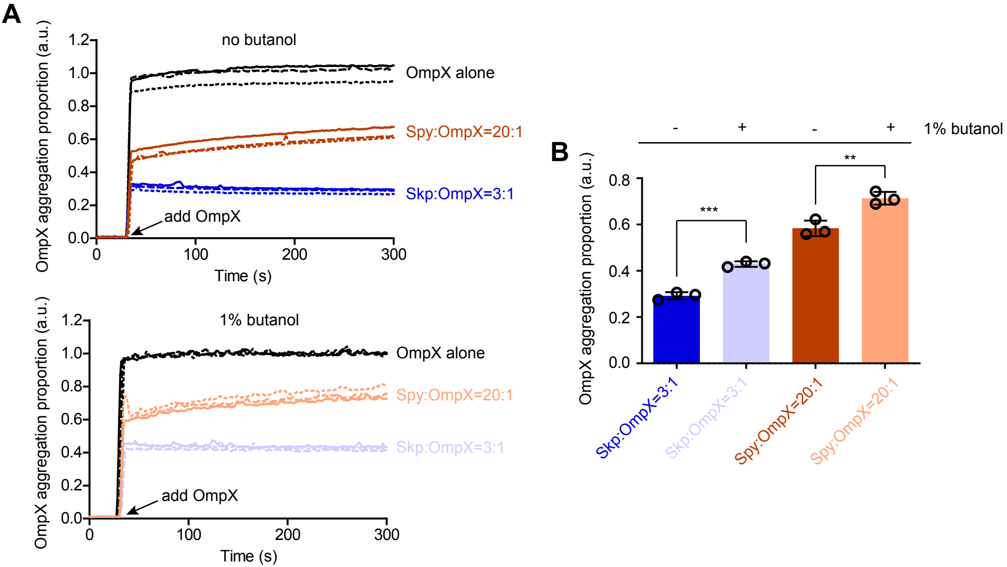

FIG S4 Spy and Skp exhibit decreased anti-aggregation activities towards OmpX in the presence of 1% butanol. (A) The anti-aggregation activities of Spy or Skp towards OmpX were monitored in the presence and absence of 1% butanol. 30 µM Spy or 4.5 µM Skp were incubated for 30 min in 40 mM HEPES, 150 mM NaCl, pH 7.5 buffer supplied with or without 1% butanol (v/v) and then OmpX (150 µM in 8 M urea) was added to a final concentration of 1.5 µM. OmpX aggregation was monitored by the light scattering signals at 380 nm. The anti-aggregation activities of Spy and Skp were expressed as the proportion of OmpX aggregation, while OmpX aggregation in the absence of any chaperone in the presence or absence of 1% butanol represented complete aggregation. (B) Both Spy and Skp showed decreased anti-aggregation activities towards OmpX when treated with 1% butanol. (mean ± SD, n = 3, individual data points are shown; unpaired two-tailed Student's t-test, **, p<0.01, ***, p<0.001).
