# Supplementary material for: Chaperone Spy Protects Outer Membrane Proteins from Folding Stress via Dynamic Complex Formation
Source: mBio. 2021 Oct 5;12(5):e02130-21. doi: 10.1128/mBio.02130-21 (PMC8546600; doi:10.1128/mBio.02130-21)
Supplement: FIG S2 [file mbio.02130-21-sf002.pdf]

FIG S2

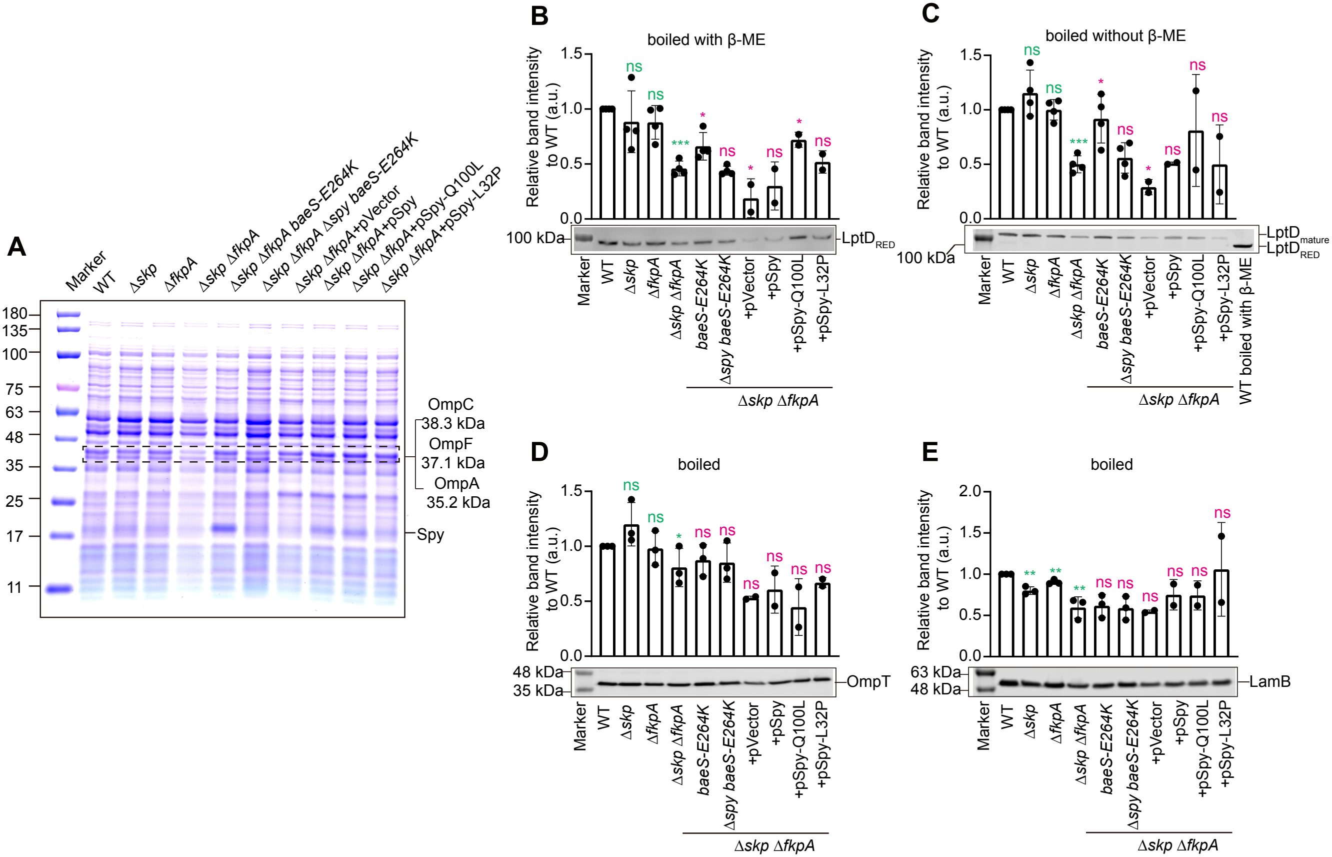

FIG S2 Overexpression of Spy partially compensates for the reduced levels of certain OMPs in the Δ*skp* Δ*fkpA* strain. (A) SDS-PAGE analysis of the whole-cell protein components of the Wild-type and chaperone deletion strains. Proteins that were deficient in the Δ*skp* Δ*fkpA* strain and were compensated by Spy overexpression were marked with a dashed box. (B, C) Immunoblotting of LptD in various strains. Before immunoblotting with an antibody against LptD, samples boiled with (B) or without (C) β-ME were separated by reducing or non-reducing SDS-PAGE, respectively. Correctly assembled LptD contains two nonconsecutive disulfide bonds and runs slower than totally reduced LptD in SDS-PAGE analysis, thereby the non-reducing SDS-PAGE enables us to detect the levels of correctly assembled LptD in various strains. (D, E) Immunoblotting of OmpT (D) and LamB (E) in the indicated strains. Samples were boiled to denature all proteins before SDS-PAGE. For B to E, quantifications and comparisons of OMP steady-state levels in various strains were displayed above the corresponding western blots. Mean ± SD, n = 2~4, individual data points are shown; unpaired two-tailed student t-test, labels in green and pink represented statistical significances of the OMP steady-state levels of indicated strains compared with the wild-type strain and the Δ*skp* Δ*fkpA* strain respectively; ns, not significant, $*P < 0.05$, $**P < 0.01$, $***P < 0.001$.
